# Interplay among p21^Waf1/Cip1^, MUSASHI-1 and Krüppel-like factor 4 in activation of *Bmi1-Cre*^*ER*^ reserve intestinal stem cells after gamma radiation-induced injury

**DOI:** 10.1038/s41598-020-75171-w

**Published:** 2020-10-27

**Authors:** Emilia J. Orzechowska, Takahito Katano, Agnieszka B. Bialkowska, Vincent W. Yang

**Affiliations:** 1 Department of Medicine, Renaissance School of Medicine at Stony Brook University, Stony Brook, NY USA; 2 Department of Molecular Biology, Faculty of Biology, University of Warsaw, Warsaw, Poland; 3 Department of Gastroenterology and Metabolism, Nagoya City University Graduate School of Medical Sciences, Nagoya, Japan; 4 Department of Physiology and Biophysics, Renaissance School of Medicine at Stony, Brook University, Stony Brook, NY USA

**Keywords:** Intestinal stem cells, Regeneration

## Abstract

Gamma radiation is a commonly used adjuvant treatment for abdominally localized cancer. Since its therapeutic potential is limited due to gastrointestinal (GI) syndrome, elucidation of the regenerative response following radiation-induced gut injury is needed to develop a preventive treatment. Previously, we showed that Krüppel-like factor 4 (KLF4) activates certain quiescent intestinal stem cells (ISCs) marked by *Bmi1-Cre*^*ER*^ to give rise to regenerating crypts following γ irradiation. In the current study, we showed that γ radiation-induced expression of p21^Waf1/Cip1^ in *Bmi1-Cre*^*ER*^ cells is likely mitigated by MUSASHI-1 (MSI1) acting as a negative regulator of p21^Waf1/Cip1^ mRNA translation, which promotes exit of the *Bmi1-Cre*^*ER*^ cells from a quiescent state. Additionally, *Bmi1*-specific *Klf4* deletion resulted in decreased numbers of MSI1^+^ cells in regenerating crypts compared to those of control mice. We showed that KLF4 binds to the *Msi1* promoter and activates its expression in vitro. Since MSI1 has been shown to be crucial for crypt regeneration, this finding elucidates a pro-proliferative role of KLF4 during the postirradiation regenerative response. Taken together, our data suggest that the interplay among p21^Waf1/Cip1^, MSI1 and KLF4 regulates *Bmi1-Cre*^*ER*^ cell survival, exit from quiescence and regenerative potential upon γ radiation-induced injury.

## Introduction

Radiation has an important role in abdominal cancer treatment, especially as an adjuvant therapy. However, gastrointestinal complications due to high-dose radiation are still a limiting factor of its usage in terms of dose and frequency^1^. Exposure of mice to a dose of 10 Gy or higher leads to acute gastrointestinal syndrome and results in high mortality rates due to extensive damage to the epithelial cells in the gastrointestinal tract. Death usually ensues within a period of approximately 4–10 days post-exposure due to bone marrow failure^2,3^. A clinically relevant dose of 12 Gy ablates proliferating cells residing within the crypt compartment, and the response of the intestinal epithelium (IE) is divided into the early and late postirradiation phases^4,5^. The early phase lasts up to 48 h post-exposure. In this period, p53-mediated apoptosis leads to crypt shrinkage or loss and shortening of the villi. In surviving cells, γ radiation induces p21^Waf1/Cip1^ expression, resulting in cell cycle arrest and activation of DNA damage repair^4–8^. At the end of the early phase, surviving cells show enhanced proliferation, which results in transient crypt fission and tissue recovery. This phase occurs from 48 to 96 h postirradiation. From 5 to 7 days post-injury, the size of the crypts and the length of the villi are restored to homeostatic (preirradiation) conditions^4,5^.

Regeneration of the IE is possible due to the presence of intestinal stem cells (ISCs), which reside at the bottom of the crypts. In homeostasis, a subpopulation of active ISCs (aISCs) produce progenitor cells that proliferate, differentiate and migrate to the top of the villi, followed by shedding into the lumen. The turnover of progenitor cells takes on average 3–5 days in the mouse small intestine^9,10^. Active ISCs exhibit high levels of Wnt activity and are more susceptible to DNA damage^11^. Following ionizing irradiation, a subpopulation of crypt cells, known as reserve ISCs (rISCs), become activated to replenish aISCs and restore tissue architecture and functions^2,12–15^. rISCs generally reside at the + 4 to + 6 position from the bottom of the crypt, are slowly cycling or quiescent and are radioresistant. To date, no single molecular marker of rISCs has been identified, although a subpopulation of the cells expresses B-cell-specific Moloney murine leukemia virus integration site 1 (BMI1)^12,16^, as demonstrated by single-cell transcriptomic analysis^17^. Despite the heterogeneous nature of the *Bmi1*-expressing population, *Bmi1-Cre*^*ER*^ cells were shown to serve as a source for tissue regeneration after γ radiation-induced gut injury^18^. Previously, we demonstrated that this process is regulated in part by Krüppel-like factor 4 (KLF4)^16,18^. KLF4 is expressed predominantly in terminally differentiated intestinal epithelial cells of the villi^19,20^. However, a few isolated nonproliferating cells located around the + 4 to + 6 position (including *Bmi1-Cre*^*ER*^ cells) also express KLF4^16,18,20^. In homeostasis, KLF4 has an antiproliferative role, and its deletion from *Bmi1*-*Cre*^*ER*^ cells resulted in increased proliferation of the *Bmi1-Cre*^*ER*^ cells. In contrast, *Bmi1-*specific *Klf4* deletion impaired the ability of *Bmi1-Cre*^*ER*^ cells to regenerate upon γ radiation-induced gut injury^16^. Therefore, KLF4 is believed to be a radioprotective factor with context-dependent functions.

MUSASHI-1 (MSI1) is an RNA-binding protein expressed in the adult small intestine and regulates post-transcriptional mRNA processing^21–28^. In homeostasis, MSI1 expression is limited to the few cells located at the bottom of the crypts. However, following ionizing radiation-induced injury, MSI1 expression was significantly elevated^27,29,30^. Increasing evidence has indicated that MSI1 and MSI2 are required for the activation of rISCs and drive exit from quiescence. Recent studies showed that mice with deletion of *Msi1* from the entire IE or *Hopx*-/*Bmi1-Cre*^*ER*^*-*marked rISCs failed to regenerate their epithelia upon γ radiation-induced injury^31^. Additionally, MSI1 and MSI2 were shown to drive mTORC1 activation, most likely through the PTEN-PIK3-AKT axis, which is required for the regenerative process^28,31^. These findings indicate the importance of the MSI1 protein as well as the *Hopx*-*Cre*^*ER*^ and *Bmi1-Cre*^*ER*^ subpopulations of rISCs in the response to γ radiation. *Hopx*- *Cre*^*ER*^ and *Bmi1-Cre*^*ER*^ indicate a largely overlapping population of rISCs; however, they are not identical. Since pre-existing experimental data focused on *Hopx*-*Cre*^*ER*^-marked rISCs^31^, we investigated the role of MSI1 in *Bmi1-Cre*^*ER*^*-*marked regenerating crypts and elucidated its function upon γ radiation-induced injury. Our results demonstrated that γ irradiation-induced expression of p21^Waf1/Cip1^ in *Bmi1-Cre*^*ER*^*-*marked cells during the early phase of the postirradiation period is retarded by MSI1, an established negative regulator of p21^Waf1/Cip1^ mRNA translation^21,25,26^. Furthermore, we elucidated the context-dependent pro-proliferative function of KLF4 upon irradiation by maintaining *Msi1* expression during the late regenerative phase. Additionally, in vitro chromatin immunoprecipitation (ChIP) analysis showed that KLF4 binds to and activates the *Msi1* promoter, suggesting a potential mechanism by which KLF4 regulates *Msi1* expression in vivo.

## Results

### p21^Waf1/Cip1^ (P21) is expressed in *Bmi1-Cre*^*ER*^*-*marked lineage cells following γ irradiation during the early phase after irradiation

Previously, we and others showed that *Bmi1-Cre*^*ER*^*-*marked cells represent one of the populations of rISCs that exit a quiescent state and start proliferating following γ irradiation to regenerate the IE^2,16,18^. To trace the changes occurring in *Bmi1-Cre*^*ER*^ marked cells, we utilized *Bmi1-Cre*^*ER*^; *Rosa26*^*eYFP*^ (*Bmi1*^*Ctrl*^) reporter mice in which eYFP was used to label *Bmi1-Cre*^*ER*^ marked rISCs and their lineages (YFP^+^ cells) upon tamoxifen injection. Duodena from nonirradiated mice or mice exposed to 12 Gy total body irradiation (TBI) were collected and analyzed according to Protocol 1 (Supplementary Fig. 1A). Previously, we observed that p21^Waf1/Cip1^ is not expressed during homeostasis and that its level is sharply induced upon injury in intestinal crypts^32^. To determine whether an increase in p21^Waf1/Cip1^ expression occurs in the *Bmi1-Cre*^*ER*^-marked cells, we performed immunofluorescence (IF) staining and analyzed the time-course expression pattern of p21^Waf1/Cip1^ in the YFP^+^ cells.

**Figure 1:**
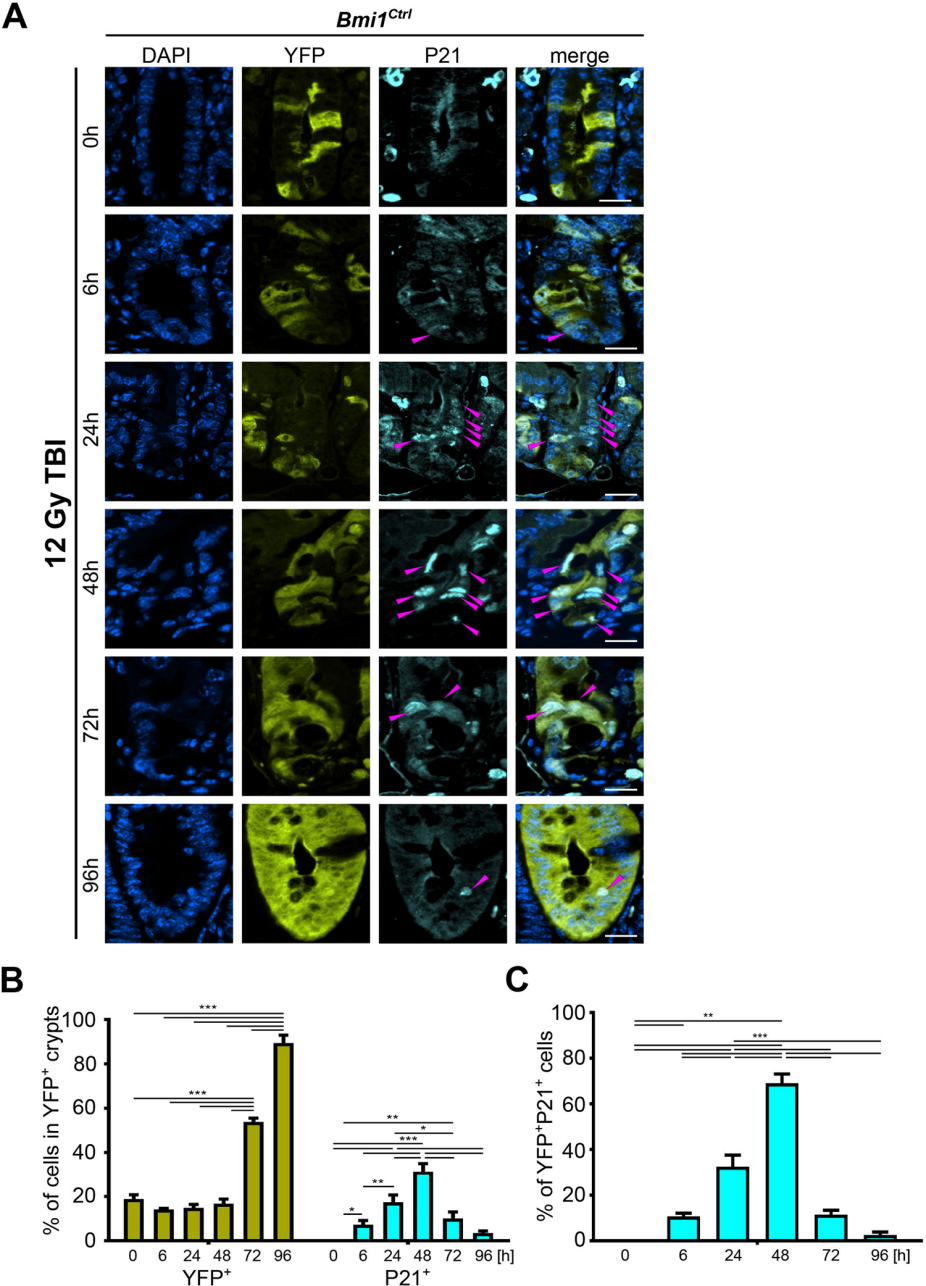
Time-dependent p21^Waf1/Cip1^ (P21) expression pattern in the YFP^+^ crypts after 12 Gy TBI of the *Bmi1*^*Ctrl*^ mice treated according to protocol 1 (Supplementary Fig. 1A). (**A**) Representative IF images of DAPI, YFP, and p21^Waf1/Cip1^ staining in the PSI crypts at 0, 6, 24, 48, 72 and 96 h after irradiation obtained under a fluorescence microscope. The scale bar represents 20 µm. P21^Waf1/Cip1+^ cells are marked by magenta arrowheads. (**B**) Quantification of the percentage of YFP^+^ or p21^Waf1/Cip1+^ cells in the YFP^+^ crypts. (**C**) Quantification of the percentage of YFP^+^p21^Waf1/Cip1+^ cells. Data are represented as the mean ± SD, 20 YFP^+^ crypts were quantified per mouse, and n = 3 mice per group. *p < 0.05, **p < 0.01 and ***p < 0.001 by one-way ANOVA.

**Figure 2:**
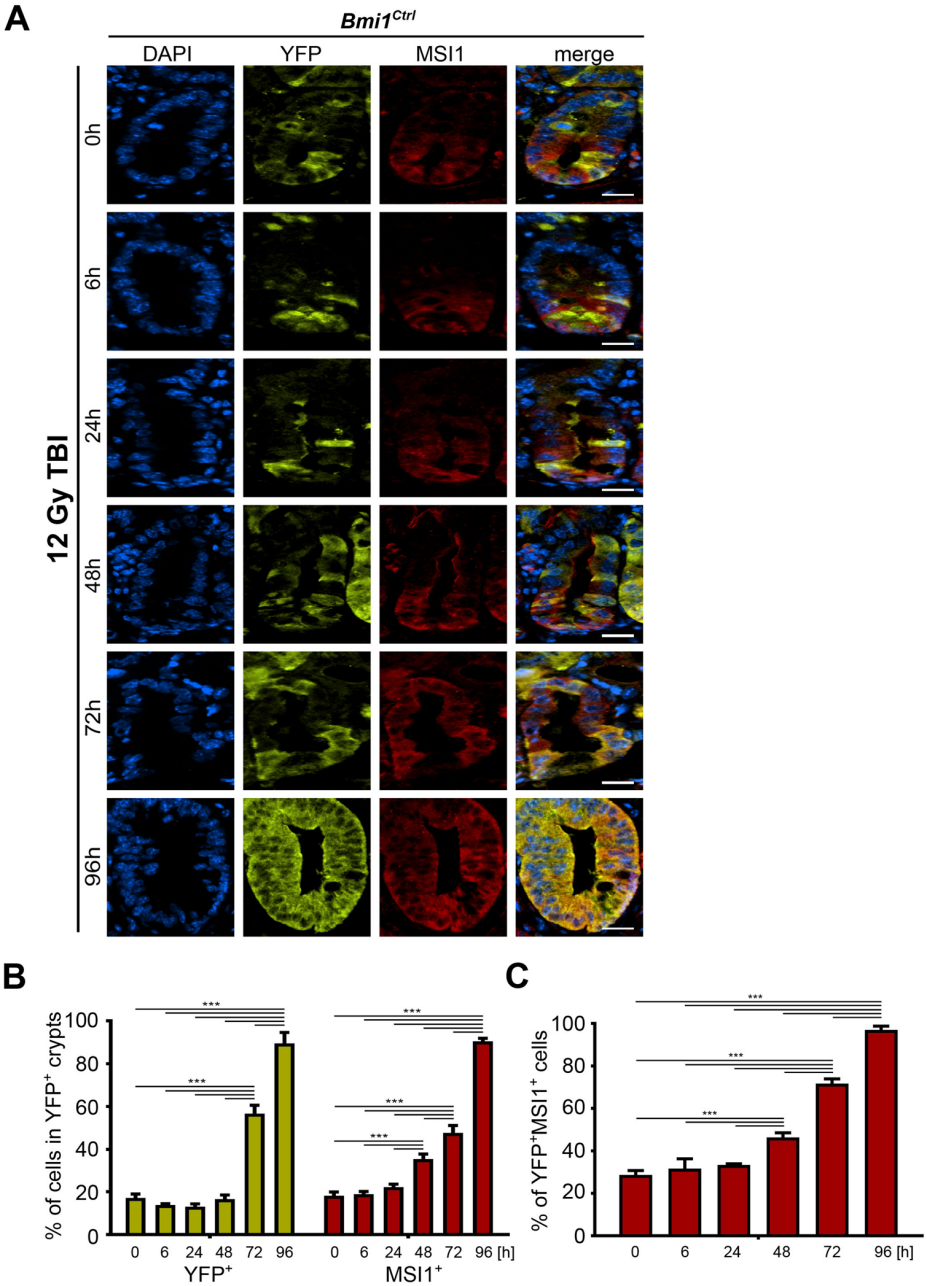
Time-dependent MSI1 expression pattern in the YFP^+^ crypts after 12 Gy TBI of the *Bmi1*^*Ctrl*^ mice treated according to protocol 1 (Supplementary Fig. 1A). (**A**) Representative IF images of DAPI, YFP, and MSI1 staining in the PSI crypts at 0, 6, 24, 48, 72 and 96 h after irradiation obtained under a fluorescence microscope. The scale bar represents 20 µm. (**B**) Quantification of the percentage of YFP^+^ or MSI1^+^ cells in the YFP^+^ crypts. (**C**) Quantification of the percentage of YFP^+^MSI1^+^ cells. Data are represented as the mean ± SD, 20 YFP^+^ crypts were quantified per mouse, and n = 3 mice per group. ***p < 0.001 by one-way ANOVA.

During homeostasis, we observed evidence of lineage tracing from the *Bmi1-Cre*^*ER*^-marked (YFP^+^) cells. Between the 0 and 96 h time points, the percentage of YFP^+^ cells doubled (Supplementary Fig. 2). Upon injury up to 48 h postirradiation, the percentage of YFP^+^ cells in the YFP^+^ crypts remained stable (Figs. 1 and 2). However, a significant increase in the percentage of YFP^+^ cells at 72 and 96 h postirradiation indicated their activation during the late postinjury phase (Figs. 1 and 2).

During homeostasis, we did not observe p21^Waf1/Cip1^ expression in the YFP^+^ cells (Supplementary Fig. 2). In contrast, 6 h postirradiation, the percentage of p21^Waf1/Cip1^-positive cells in the YFP^+^ crypts started increasing, especially in the transient-amplifying (TA) zone, and peaked 48 h post-injury (Figs. 1A and 1B). Simultaneously, we observed that the percentage of YFP^+^ cells coexpressing p21^Waf1/Cip1^ increased and peaked 48 h postirradiation (Fig. 1C). In contrast, during the late postinjury phase, the expression of p21^Waf1/Cip1^ both in the YFP^+^ crypts and in the YFP^+^ cells started to decrease. Ninety-six hours postirradiation, the percentages of cells in the YFP^+^ crypts or the YFP^+^ cells expressing p21^Waf1/Cip1^ were infinitesimal (Fig. 1). Taken together, these data showed that the p21^Waf1/Cip1^ protein levels are increased in response to γ radiation-induced injury, and at the 48 h time point, the majority of the YFP^+^ cells coexpressed p21^Waf1/Cip1^.

### MSI1 is expressed in YFP^+^ cells following γ irradiation during the late phase after irradiation

MSI1 expression is required for the activation of rISCs, their exit from quiescence, and cell cycle entry after γ radiation-induced injury^31^. First, to assess the role of MSI1 in the regeneration of YFP^+^ cells, we performed a time course analysis of MSI1 expression in the sham and irradiated *Bmi1*^*Ctrl*^ mice. We determined that during homeostasis, MSI1 is expressed by a small number of cells located at the bottom of the YFP^+^ crypts and that some of them were YFP^+^ cells (Supplementary Fig. 3). A similar percentage of cells expressed MSI1 up to 24 h postirradiation (Fig. 2). By contrast, we observed that at 48 h postirradiation, the level of MSI1 started to increase in all cells in the YFP^+^ crypts, including the YFP^+^ cells themselves (Fig. 2). The expression levels continued to rise during the late post-injury phase and peaked at 96 h postirradiation. Taken together, these data showed that MSI1 expression is correlated with the regenerative potential of the YFP^+^ cells, which is consistent with previously published data reporting radiation-induced MSI1 expression^27,29,30^.

### MSI1 is a negative regulator of *Cdkn1a* mRNA translation

Since we demonstrated that p21^Waf1/Cip1^ was expressed predominantly up to 48 h post-injury and diminished thereafter, while MSI1 expression increased, we surmised that MSI1 may regulate the expression of p21^Waf1/Cip1^ when cells enter the late postinjury phase (Figs. 1 and 2). To focus on early lineages marked by YFP, we utilized a different treatment protocol that allowed the capture of this specific cell population (Protocol 2; Supplementary Fig. 1B). Analysis of the expression patterns of p21^Waf1/Cip1^ and MSI1 in the control group confirmed the results obtained from Protocol 1 (Supplementary Fig. 4). After γ irradiation, p21^Waf1/Cip1^ expression peaked at 48 h and started to diminish during the late postirradiation phase, along with increased expression of MSI1 (Supplementary Fig. 5). Importantly, we observed that the percentage of YFP^+^P21^+^MSI1^+^ cells during the late postinjury phase decreased significantly between 48 and 96 h postirradiation, similar to the YFP^+^P21^+^ cells. In contrast, the population of YFP^+^MSI1^+^ cells steadily increased (Supplementary Fig. 5C). Statistical analysis showed a significant negative correlation between p21^Waf1/Cip1^ and MSI1 expression in the YFP^+^ crypts (Supplementary Fig. 5D), suggesting that MSI1 may mitigate p21^Waf1/Cip1^ expression.

**Figure 3:**
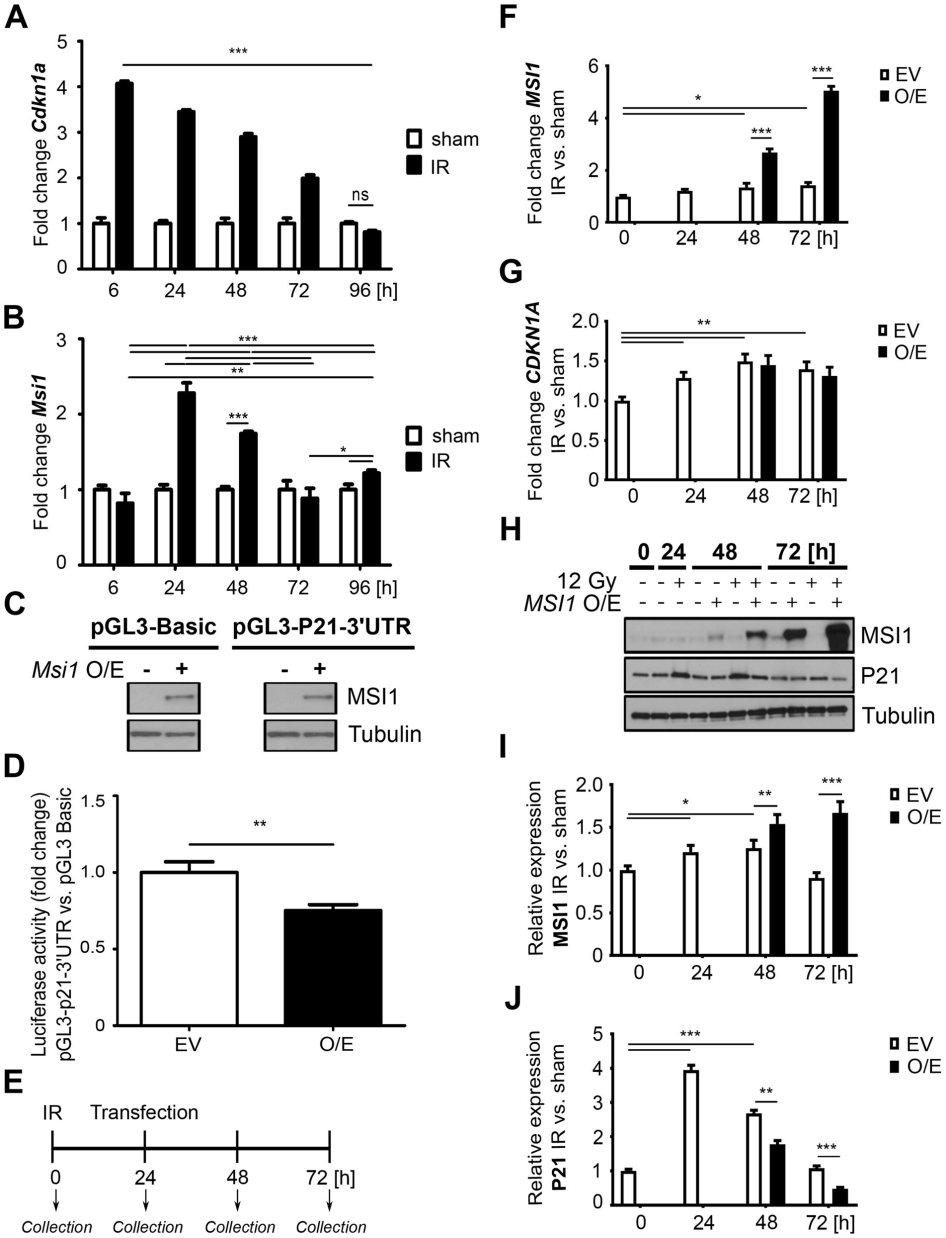
MSI1 is a negative regulator of *p21*^*Waf1/Cip1*^ (*Cdkn1a*) mRNA translation. (**A**, **B**) qRT-PCR analysis of *Cdkn1a* (**A**) and *Msi1* (**B**) in the FACS-sorted YFP^+^ cells isolated from the *Bmi1*^*Ctrl*^ mice treated according to protocol 1 (Supplementary Fig. 1A). The sham mice were used as a control. IR, irradiated group (12 Gy TBI). Data are represented as the mean ± SD, n = 3 mice per group. *p < 0.05, **p < 0.01 and p < 0.001 by one-way ANOVA. (**C**, **D**) Luciferase assays of HEK293T cells transfected with the pGL3 Basic or pGL3-p21^Waf1/Cip1^ 3′-UTR vector and *Msi1* overexpression vector (pCMV6-AC-GFP-Msi1). O/E, overexpression. EV, empty vector. (**C**) Western blot analysis of *Msi1* overexpression. Full-length blots are presented in the “Supplementary file”. (**D**) Relative luciferase activity. Data are represented as the mean ± SD, n = 3. **p < 0.01 by Student’s t-test. (**E**–**J**) Analysis of the effect of MSI1 on *p21*^*Waf1/Cip1*^ expression after γ irradiation-induced injury in vitro. HEK293T cells were seeded on a plate and irradiated with a total dose of 0 (sham) or 12 Gy (IR, irradiated), and 24 h later, *MSI1* was overexpressed. Cells were collected at 0, 24, 48 and 72 h. O/E, overexpression. EV, empty vector. (**E**) Experimental outline. (**F**, **G**) qRT-PCR analysis of *MSI1* (**F**) and *CDKN1A* (**G**) in HEK293T cells. (**H**) Western blot analysis of MSI1 and p21^Waf1/Cip1^ (P21) protein expression in HEK293T cells. Full-length blots are presented in the “Supplementary file”. (**I**, **J**) Densitometric analysis of protein expression in HEK293T cells performed using ImageJ software. Data are represented as the mean ± SD, n = 3. *p < 0.05, **p < 0.01 and ***p < 0.001 by Student’s t-test.

To further elucidate the interaction between p21^Waf1/Cip1^ and MSI1, we sorted the YFP^+^ cells obtained from the *Bmi1*^*Ctrl*^ mice treated according to Protocol 1 (Supplementary Fig. 1A) by fluorescence-activated cell sorting (FACS) analysis and performed qRT-PCR analysis of *Cdkn1a* and *Msi1* gene expression. We observed a significant increase in *Cdkn1a* expression compared to that in the nonirradiated mice and a progressive decrease over time (Fig. 3A). In contrast, the *Msi1* levels increased later (24 h postirradiation), decreased by 72 h to baseline and then slightly increased at the end of the late postinjury phase compared to those of the nonirradiated control (Fig. 3B). MSI1 is an RNA-binding protein and was previously reported to act as a translational suppressor of *Cdkn1a* mRNA expression^2,21,25,33–42^. Therefore, we performed a luciferase assay to confirm that MSI1 negatively regulates *Cdkn1a* gene expression. We transfected HEK293T-GFP cells with pGL3-Basic or pGL3-*P21-3′UTR* reporter vectors containing luciferase linked to the 3′ untranslated region (UTR) of the *Cdkn1a* mRNA. The cells were also transfected with an expression vector encoding *Msi1* or an empty vector as a control. First, we confirmed similar levels of MSI1 by Western blot analysis of the cells transfected with the MSI1 expression vector (Fig. 3C). As expected, a luciferase assay showed that *Msi1* overexpression significantly reduced the reporter activity of the 3′UTR of *Cdkn1a* compared to that of the control vector (Fig. 3D), consistent with existing data^2,21,25,33–42^.

Next, to examine whether MSI1 negatively regulates *Cdkn1a* expression following injury, we irradiated HEK293T-GFP cells and 24 h later overexpressed *MSI1* or transfected cells with an empty control vector (Fig. 3E). We performed qRT-PCR and Western blot analysis of p21^Waf1/Cip1^ and MSI1. The results showed that the *MSI1* levels were increased compared to those of the empty vector group (Fig. 3F). We also observed a slight induction of endogenous *MSI1* expression due to irradiation at 48 and 72 h post-injury compared to that at 0 h (Fig. 3F, white bars). Moreover, we showed that the endogenous *CDKN1A* levels were significantly increased postirradiation compared to those at 0 h, and *MSI1* overexpression did not affect the *CDKN1A* levels (Fig. 3G). This observation is consistent with a previous finding showing that MSI1 regulates target genes at the translational level^27–29^. Therefore, we performed Western blot analysis of p21^Waf1/Cip1^ and MSI1 (Fig. 3H). We demonstrated that there was a slight increase in the endogenous MSI1 levels at 24 and 48 h compared to 0 h due to irradiation (Fig. 3I, white bars). Overexpression of *MSI1* resulted in significantly increased levels at the 48 and 72 h time points compared to those of the empty vector control group (Fig. 3I). Furthermore, the levels of endogenous p21^Waf1/Cip1^ were increased compared to those at the 0 h time point after irradiation and gradually decreased over time (Fig. 3I, white bars). Importantly, due to *MSI1* overexpression, the total p21^Waf1/Cip1^ protein level was significantly reduced at 48 h and 72 h post-injury (Fig. 3J) compared to that of the empty vector control groups. Taken together, the results obtained from the in vitro experiments showed that MSI1 negatively regulates p21^Waf1/Cip1^ expression at the translational level during homeostasis, as well as upon injury. Given that the analysis of p21^Waf1/Cip1^ and MSI1 coexpression in the YFP^+^ cells in vivo also demonstrated a negative correlation between these two proteins, the in vitro study suggests that this mechanism may underlie the activation of YFP^+^ cells upon irradiation-induced injury.

### KLF4 sustains *Msi1* expression during the late postirradiation phase

**Figure 4:**
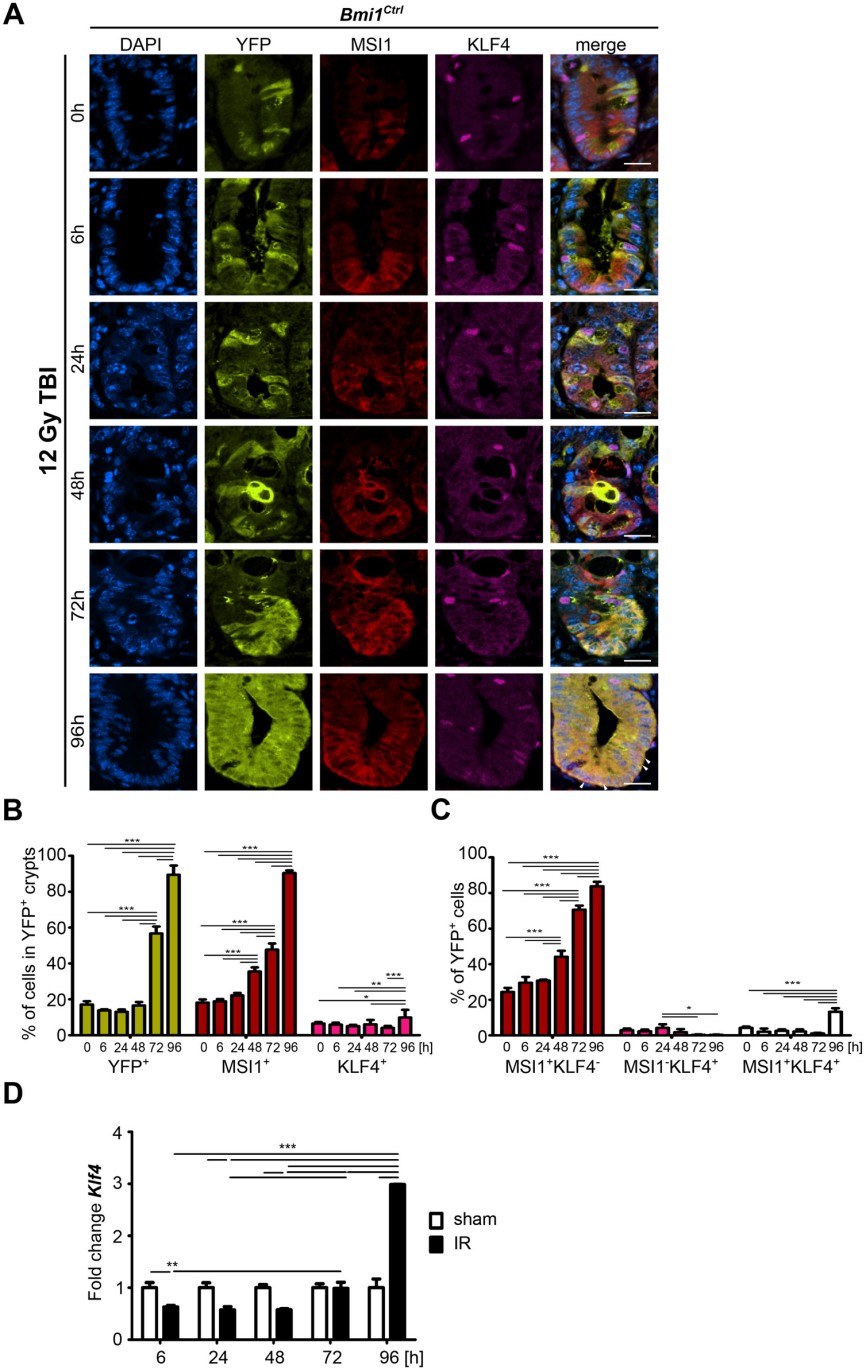
Time-dependent MSI1 and KLF4 expression patterns in the YFP^+^ crypts after 12 Gy TBI of the *Bmi1*^*Ctrl*^ mice treated according to protocol 1 (Supplementary Fig. 1A). (**A**) Representative IF images of DAPI, YFP, MSI1, and KLF4 staining in the PSI crypts at 0, 6, 24, 48, 72 and 96 h after irradiation obtained under a fluorescence microscope. The scale bar represents 20 µm. (**B**) Quantification of the percentage of YFP^+^, MSI1^+^ or KLF4^+^ cells in the YFP^+^ crypts. (**C**) Quantification of the percentage of YFP^+^ cells costained with MSI1, KLF4 or MSI1 and KLF4 together. Data are represented as the mean ± SD, 20 YFP^+^ crypts were quantified per mouse, and n = 3 mice per group. *p < 0.05, **p < 0.01 and ***p < 0.001 by one-way ANOVA. (**D**) qRT-PCR analysis of *Klf4* expression in the FACS-sorted YFP^+^ cells isolated from the *Bmi1*^*Ctrl*^ mice treated according to protocol 1 (Supplementary Fig. 1A) at 0, 6, 24, 48, 72 and 96 h after irradiation. The sham mice were used as a control. IR, irradiated group (12 Gy TBI). Data are represented as the mean ± SD, n = 3 mice per group. *p < 0.05, **p < 0.01 and ***p < 0.001 by one-way ANOVA.

**Figure 5:**
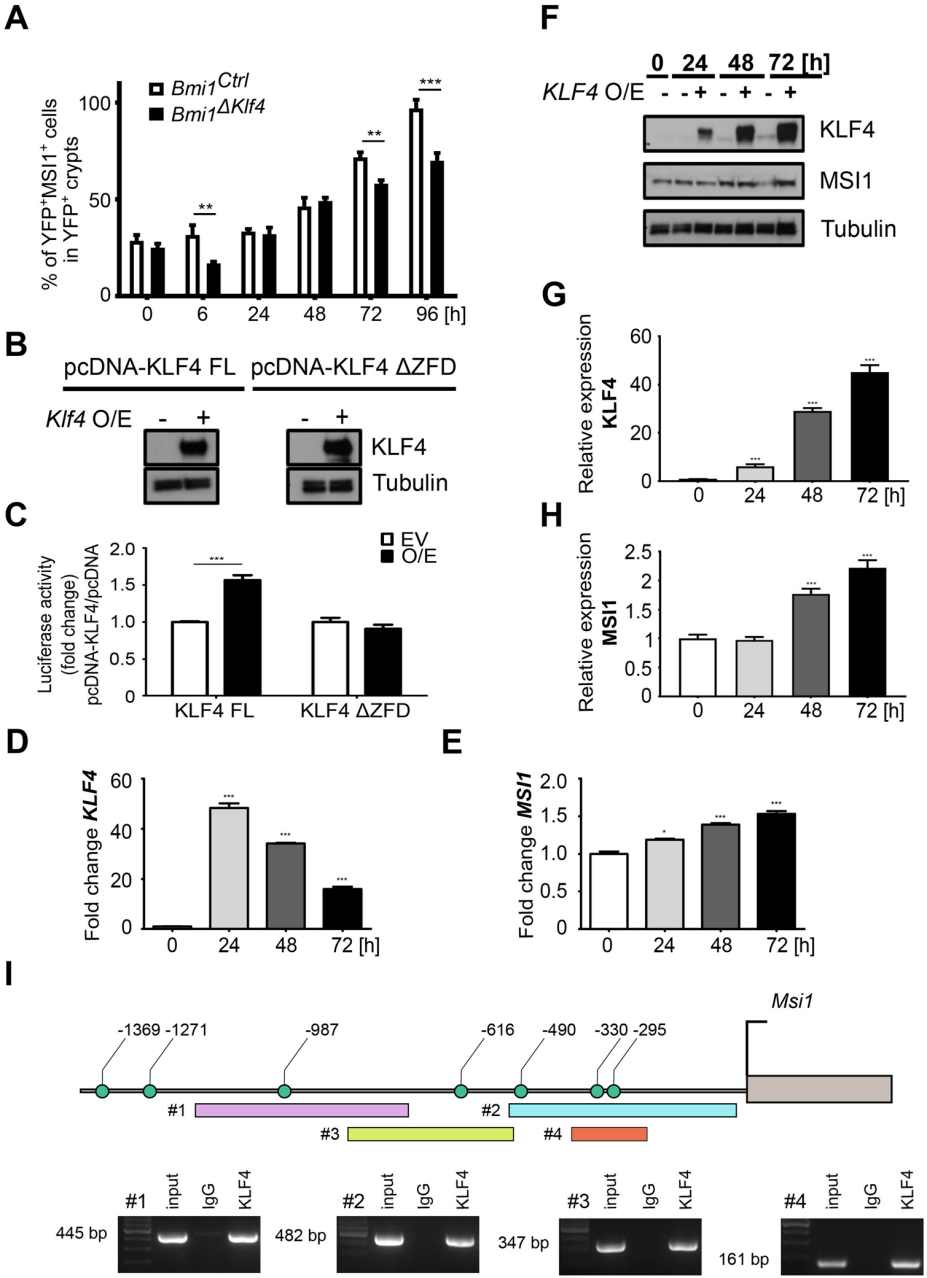
KLF4 positively regulates *MSI1* expression by directly binding to its promoter. (**A**) Comparison of the percentage of YFP^+^MSI1^+^ cells in the YFP^+^ crypts of the *Bmi1*^*Ctrl*^ and *Bmi1*^*∆Klf4*^ mice at 0, 6, 24, 48, 72 and 96 h after irradiation based on IF staining analysis. Data are represented as the mean ± SD, 20 YFP^+^ crypts were quantified per mouse, and n = 3 mice per group. **p < 0.01 and ***p < 0.001 by one-way ANOVA. (**B**, **C**) Luciferase assays of the HEK293T cells transfected with pcDNA-*Klf4* FL or pcDNA-*Klf4* ΔZFD mutant vector and pEZX-PG02 containing the *Msi1* promoter sequence. O/E, overexpression. EV, empty vector. (**B**) Western blot analysis of KLF4. Full-length blots are presented in the “Supplementary file”. (**C**) Relative luciferase activity. Data are represented as the mean ± SD, n = 3. ***p < 0.001 by Student’s t-test. (**D**–**H**) Analysis of the effect of KLF4 FL on *MSI1* expression in vitro. O/E, overexpression. EV, empty vector. (**D**, **E**) qRT-PCR analysis of *KLF4* (**D**) and *MSI1* (**E**) expression in HEK293T cells. Cells collected at 0 h were used as controls. (**F**) Western blot analysis of KLF4 and MSI1 in HEK293T cells. Full-length blots are presented in the “Supplementary file”. (**G**, **H**) Densitometric analysis of KLF4 (**G**) and MSI1 (**H**) in HEK293T cells was performed using ImageJ software. Data are represented as the mean ± SD, n = 3. *p < 0.05, **p < 0.01 and ***p < 0.001 by Student’s t-test. (**I**) ChIP-PCR analysis of KLF4 binding to the *Msi1* promoter. Schematic represents the − 1.4-kb region upstream of the *Msi1* TSS showing potential KLF4 binding sites (green circles). ChIP-PCR primer locations are marked with rectangles. DNA electrophoresis gels show PCR products obtained after the reaction with ChIP-purified DNA. Rabbit IgG was used as a negative control. Full-length blots are presented in the “Supplementary file”.

Recently, we reported that regenerative potential of *Bmi1-Cre*^*ER*^*-*marked cells following γ irradiation is regulated in part by KLF4. We also showed that KLF4 is an antiproliferative transcription factor that becomes pro-proliferative during regeneration^16^. Since MSI1 also stimulates regeneration of the IE after γ irradiation-induced injury^31^ (Fig. 2, Supplementary Fig. 5), we examined whether KLF4 and MSI1 interact. We performed time course analysis of MSI1 and KLF4 expression in samples collected from the *Bmi1*^*Ctrl*^ mice after 12 Gy TBI according to Protocol 1 (Supplementary Fig. 1A). In homeostasis, some YFP^+^ cells coexpressed MSI1, but only a few were YFP^+^MSI1^+^KLF4^+^ (Supplementary Fig. 3). After γ irradiation-induced injury, we initially observed a decrease in KLF4 expression in the YFP^+^ crypts and an increase at 96 h postirradiation (Fig. 4A–C). Similarly, analysis of MSI1 and KLF4 coexpression in the YFP^+^ cells showed that between 0 and 72 h postirradiation, a very small percentage of YFP^+^ cells coexpressed MSI1 and KLF4. By contrast, this subpopulation increased significantly 96 h postirradiation (Fig. 4C). Furthermore, qRT-PCR analysis of *Klf4* expression in the FACS-sorted YFP^+^ cells obtained from the *Bmi1*^*Ctrl*^ mice showed that up to 72 h post-injury, *Klf4* expression was either decreased or not changed compared to that in the sham mice. However, at 96 h postirradiation, *Klf4* expression was significantly increased (Fig. 4D). These observations suggested a putative interaction between MSI1 and KLF4 96 h post-injury. Therefore, we analyzed the effect of *Bmi1-*specific *Klf4* deletion on *Msi1* expression during injury-induced regeneration, and we utilized *Bmi1*^*∆Klf4*^ mice in combination with Protocol 1 (Supplementary Fig. 1A). In this animal model, tamoxifen-induced Cre-mediated recombination resulted not only in labeling these cells with YFP protein but also in *Bmi1*-specific deletion of the *Klf4* gene. In homeostasis, similar to the results obtained for the *Bmi1*^*Ctrl*^ mice, we observed stable and low levels of MSI1 expression in both the YFP^+^ crypts and the YFP^+^ cells (Supplementary Fig. 6). Upon irradiation, we observed an increase in MSI1 expression in the *Bmi1*^*∆Klf4*^ mice similar to that observed in the *Bmi1*^*Ctrl*^ mice (Supplementary Fig. 7, Fig. 2, and Fig. 4). However, both the percentage of YFP^+^ cells in the YFP^+^ crypts and the percentage of YFP^+^ cells coexpressing MSI1 were significantly decreased in the *Bmi1*^*∆Klf4*^ mice compared to the *Bmi1*^*Ctrl*^ mice (Figs. 4 and 5A and Supplementary Fig. 7).

Since we observed a decreased number of YFP^+^ cells expressing *Msi1* upon injury in the *Klf4*-deleted *Bmi1-Cre*^*ER*^*-*marked cells, we hypothesized that KLF4 positively regulates *Msi1* expression. To address this hypothesis, we performed a luciferase assay using HEK293T-GFP cells cotransfected with a vector containing the *Gaussia luciferase* gene driven by the mouse *Msi1* promoter and with a plasmid encoding the mouse full-length *Klf4* or mouse *Klf4* gene with deletion of the C-terminal DNA-binding domain. Control cells were transfected with the empty vector. First, we confirmed *Klf4* overexpression by Western blot analysis (Fig. 5B). Notably, a luciferase assay showed that overexpression of full-length *Klf4* increased the relative activity of the *Msi1* promoter compared to that of the empty vector control, while the *Klf4* mutant with deletion of the C-terminal DNA-binding domain did not affect this activity (Fig. 5C).

To further elucidate the effect of KLF4 on *MSI1* expression, we overexpressed full-length human *KLF4* in HEK293T-GFP cells and analyzed total *MSI1* expression at both the mRNA and protein levels. First, we confirmed the overexpression of *KLF4* as shown by the total *KLF4* mRNA levels (Fig. 5D). Moreover, we observed increased *MSI1* expression levels (Fig. 5E). Additionally, the significantly increased KLF4 protein levels (Fig. 5F,G) mirrored the increased levels of MSI1 protein (Fig. 5F,H).

Finally, we examined whether KLF4 directly regulates *Msi1* promoter activity. We performed ChIP-PCR analysis using HEK293T-GFP cells cotransfected with a vector encoding the mouse *Msi1* promoter (up to − 1.5 kb) and a plasmid encoding full-length mouse *Klf4*. We identified 7 putative KLF4 binding sites within the mouse *Msi1* promoter and found that KLF4 binds to the regions located at positions − 295, − 330, − 616 and − 987 bp (Fig. 5I). KLF4 may also bind to the − 490 bp position. However, due to the high GC content in this region, we could not confirm this hypothesis. Taken together, these results demonstrated that KLF4 is a positive regulator of *Msi1* expression by directly binding to *cis*-elements of its promoter. These findings also suggested that this mechanism may occur in vivo since *Bmi1*^*∆Klf4*^ mice present lower *Msi1* expression than *Bmi1*^*Ctrl*^ mice.

### KLF4 positively regulates the regenerative potential of the YFP^+^ cells during regeneration upon γ irradiation-induced injury

**Figure 6:**
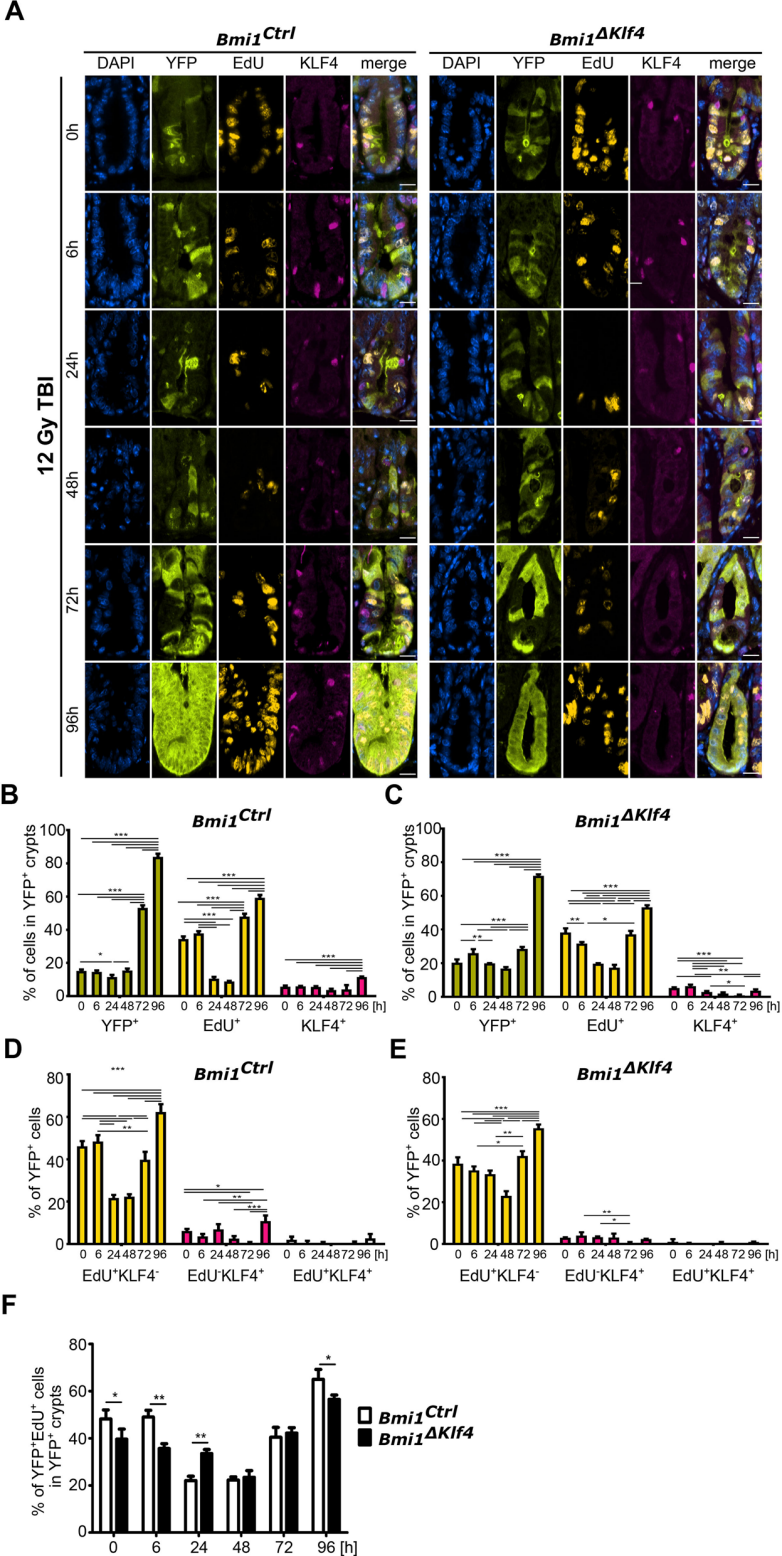
KLF4 influences the proliferative ability of cells in YFP^+^ crypts after 12 Gy TBI of the *Bmi1*^*Ctrl*^ and *Bmi1*^*∆Klf4*^ mice treated according to protocol 1 (Supplementary Fig. 1A). (**A**) Representative IF images of DAPI, YFP, EdU, and KLF4 staining in the PSI crypts at 0, 6, 24, 48, 72 and 96 h after irradiation obtained under a fluorescence microscope. The scale bar represents 20 µm. (**B**, **C**) Quantification of the percentage of YFP^+^, EdU^+^ or KLF4^+^ cells in the YFP^+^ crypts of the *Bmi1*^*Ctrl*^ (**B**) and *Bmi1*^*∆Klf4*^ (**C**) mice. (**D**, **E**) Quantification of the percentage of YFP^+^ cells costained with EdU, KLF4 or EdU and KLF4 together in the *Bmi1*^*Ctrl*^ (**D**) and *Bmi1*^*∆Klf4*^ (**E**) mice. (**F**) Comparison of the percentage of YFP^+^EdU^+^ cells in the YFP^+^ crypts of the *Bmi1*^*Ctrl*^ and *Bmi1*^*∆Klf4*^ mice. Data are represented as the mean ± SD, 20 YFP^+^ crypts were quantified per mouse, and n = 3 mice per group. *p < 0.05, **p < 0.01 and ***p < 0.001 by one-way ANOVA.

As presented above, *Klf4* expression affected both *Msi1* expression and lineage tracing of the YFP^+^ cells (Figs. 4 and 5A and Supplementary Fig. 7). Therefore, we analyzed the proliferative potential of the YFP^+^ cells in the *Bmi1*^*Ctrl*^ and *Bmi1*^*∆Klf4*^ mice treated according to Protocol 1 (Supplementary Fig. 1A). Our results showed that during homeostasis, the percentage of YFP^+^ cells doubled over a 96 h period in both the *Bmi1*^*Ctrl*^ and *Bmi1*^*∆Klf4*^ mice. Additionally, a similar percentage of cells in the YFP^+^ crypts incorporated 5-ethynyl-2′-deoxyuridine (EdU) in both the *Bmi1*^*Ctrl*^ and *Bmi1*^*∆Klf4*^ mice. We observed that KLF4 expression was reduced in the YFP^+^ crypts of the *Bmi1*^*∆Klf4*^ mice compared to the *Bmi1*^*Ctrl*^ mice (Supplementary Fig. 8A–C). The percentage of EdU^-^KLF4^+^ cells was relatively stable in the *Bmi1*^*Ctrl*^ mice compared to the *Bmi1*^*∆Klf4*^ mice (Supplementary Fig. 8D,E). Furthermore, analysis of the YFP^+^ cells showed that over the time course, the percentage of cells incorporating EdU (subpopulations of EdU^+^KLF4^−^ and EdU^+^KLF4^+^) slightly decreased in the *Bmi1*^*Ctrl*^ mice but significantly increased in the *Bmi1*^*∆Klf4*^ mice at the 96 h time point (Supplementary Fig. 8A,D–F). These data confirmed that in homeostasis, KLF4 plays an antiproliferative role^16^. After γ irradiation-induced injury in both the *Bmi1*^*Ctrl*^ and *Bmi1*^*∆Klf4*^ mice, we performed lineage tracing, and the percentage of YFP^+^ cells increased (Fig. 6A–C). EdU incorporation either in the YFP^+^ crypts or the YFP^+^ cells (subpopulations of EdU^−^KLF4^+^ and EdU^+^KLF4^+^ cells) was significantly decreased between 0 and 48 h post-injury in both the *Bmi1*^*Ctrl*^ mice (Fig. 6A,B,D) and *Bmi1*^*∆Klf4*^ mice (Fig. 6A,C,E). However, during the late postinjury phase, we observed an increase in EdU incorporation in both the *Bmi1*^*Ctrl*^ (Fig. 6A,B,D) and *Bmi1*^*∆Klf4*^ mice (Fig. 6A,C,E). Additionally, we observed that at 96 h postirradiation, the KLF4 expression level in the YFP^+^ crypts or the YFP^+^ cells (subpopulations of EdU^+^KLF4^−^ and EdU^+^KLF4^+^ cells) increased in the *Bmi1*^*Ctrl*^ mice compared to the *Bmi1*^*∆Klf4*^ mice (Fig. 6A–E). Taken together, these data showed that even though EdU incorporation at 48 h postirradiation was reduced to a similar extent in both the *Bmi1*^*Ctrl*^ and *Bmi1*^*∆Klf4*^ mice, the *Bmi1*^*Ctrl*^ mice had better regenerative ability than the *Bmi1*^*∆Klf4*^ mice at 96 h postirradiation (Fig. 6F).

**Figure 7:**
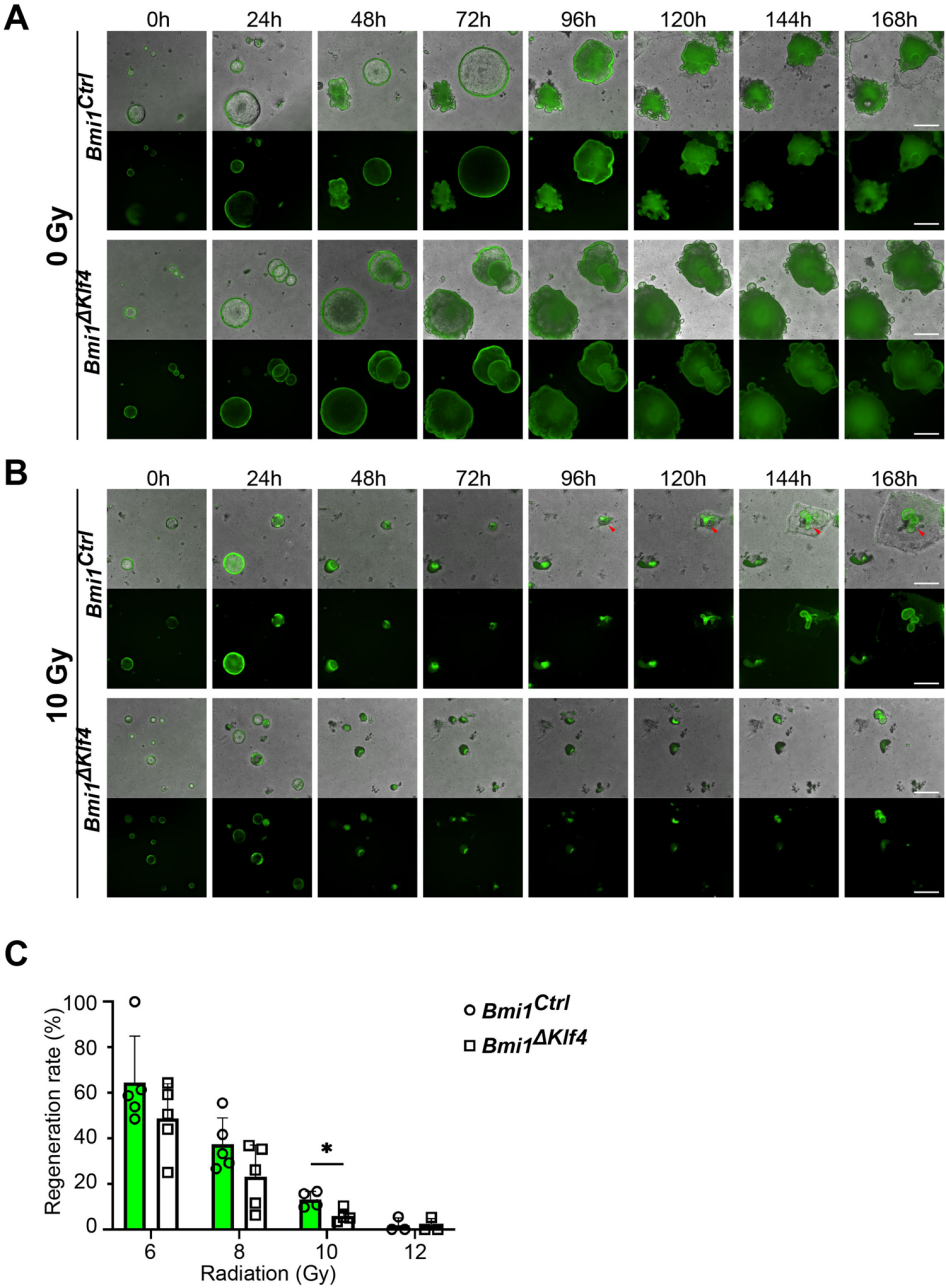
KLF4 influences YFP^+^-derived organoid formation and regenerative capability in response to γ radiation-induced injury. (**A**, **B**) Representative images of organoids derived from the FACS-sorted YFP^+^ cells isolated from the *Bmi1*^*Ctrl*^ and *Bmi1*^*∆Klf4*^ mice at 0, 6, 24, 48, 72, 96, 120, 144 and 168 h after irradiation obtained under a fluorescence microscope. Regenerating organoids are marked by red arrowheads. The lower panel represents fluorescent images, and the upper panel represents merged images of bright-field and fluorescent images. The scale bar represents 500 µm. (**C**) Quantification of the regeneration rate of organoids derived from the *Bmi1*^*Ctrl*^ and *Bmi1*^*∆Klf4*^ mice exposed to 6, 8, 10 and 12 Gy γ irradiation. Data are represented as the mean ± SD, n = 4–5 mice per group. *p < 0.05 by one-way ANOVA.

To further elucidate the role of KLF4 in the *Bmi1-Cre*^*ER*^*-*marked cells that drive regeneration, we established an ex vivo organoid model derived from the FACS-sorted YFP^+^ cells isolated from the *Bmi1*^*Ctrl*^ and *Bmi1*^*∆Klf4*^ mice. We exposed the organoids to different doses of γ irradiation. There was no difference in the growth of the nonirradiated organoids obtained from the *Bmi1*^*Ctrl*^ and *Bmi1*^*∆Klf4*^ mice (Fig. 7A,C). After exposure to γ irradiation, the organoids in both groups shrunk within the first 48 h and started to regenerate between 48 and 96 h postirradiation. The regeneration rate of the organoids decreased with the increase in radiation dose in both groups (Fig. 7, Supplementary Fig. 9). However, the overall regeneration rate of the organoids derived from the *Bmi1*^*Ctrl*^ mice was higher than that of the organoids derived from the *Bmi1*^*∆Klf4*^ mice, with a significant difference observed at the dose of 10 Gy (Fig. 7C).

Additionally, we performed an analysis of YFP^+^ crypt survival. We observed that the ratio of YFP^+^ proliferating crypts after irradiation vs. with the sham treatment in the *Bmi1*^*Ctrl*^ mice was higher than that in the *Bmi1*^*ΔKlf4*^ mice (Supplementary Fig. 10). Taken together, these data showed that the *Bmi1-Cre*^*ER*^*-*marked cell-driven regenerative potential upon γ irradiation-induced injury is reduced in the absence of KLF4 and that the ex vivo model reflects changes observed in vivo*.*

## Discussion

According to a hierarchical model, active ISCs serve as a source of constant replenishment of cells in homeostasis, and rISCs become activated following depletion of aISCs upon injury^12–14^. Recent studies have shown that regeneration of the IE is a complex process driven by numerous subpopulations of rISCs, as well as more differentiated cells due to the high plasticity of the cells residing in the crypts^15,16,43–46^. *Bmi1-Cre*^*ER*^*-*marked cells represent an important group of rISCs that contribute to the regeneration of IE^16,18,47^. Additionally, Tian et al. demonstrated that lineage tracing originating from *Bmi1*-GFP cells after injury was significantly increased compared to that under homeostasis, which is consistent with the characteristics of a slowly cycling rISC population at steady state that serves as a source of regeneration upon injury^13^.

In the current study, we utilized tamoxifen-induced *Bmi1*^*Ctrl*^ and *Bmi1*^*∆Klf4*^ mouse models to focus on *Bmi1-Cre*^*ER*^*-*marked cells and their immediate progeny (YFP^+^ cells) and addressed the role of KLF4 in the post radiation-induced regenerative response. Given that tamoxifen administration may influence IE physiology, both the irradiated and sham-treated mice were injected with tamoxifen^48,49^.

We previously showed that upon irradiation, the expression of p21^Waf1/Cip1^ was increased in the crypt compartment^31^. P21^Waf1/Cip1^ is a cyclin-dependent kinase inhibitor, and elevation of this molecule may result in cell cycle arrest in both G1/S and G2/M phases^50–55^. We observed here that upon injury, the majority of YFP^+^ cells, especially at the end of the early postinjury phase (48 h), expressed p21^Waf1/Cip1^ protein (Fig. 1 and Supplementary Fig. 5), which confirms previous findings that cell cycle arrest protects these cells from apoptosis induced by radiation injury. However, to serve as a source of regeneration, these cells need to enter the cell cycle. Here, we demonstrated that exit from cell cycle arrest may be achieved by inhibiting *p21*^*Waf1/Cip1*^ mRNA translation by MSI1 (Fig. 3). MSI1 is an RNA-binding protein member of the MUSASHI RNA-binding protein family and has been shown to regulate the cell cycle, cell proliferation, cell differentiation, and apoptosis^21–23,27^. Previously published in vitro studies showed that MSI1 is crucial to maintain cells in a proliferative state, most likely due to repression of translation of mRNA encoding proteins that inhibit cell cycle progression, including p21^Waf1/Cip1^^21,24–26,35–42^. In vivo, MSI1 was described as a stem cell marker with very low expression in homeostasis that significantly increases upon irradiation injury^27,29,30^. Interestingly, MSI1 was shown to be indispensable for crypt regeneration upon irradiation-induced injury (including *Bmi1-Cre*^*ER*^ marked cells), and rISCs with MSI protein ablation were blocked in G1 (not G0) phase and were not able to enter S-phase in response to 12 Gy TBI^31^. Here, we confirmed that MSI1 binds directly to the mouse *p21*^*Waf1/Cip1*^ 3′UTR in vitro and acts as a translational suppressor of *p21*^*Waf1/Cip1*^ mRNA (Fig. 3C,D). We also showed that MSI1 decreases p21^Waf1/Cip1^ expression at the translational level upon irradiation in vitro (Fig. 3E–J). Analysis of IF staining of mouse tissues showed a negative correlation between p21^Waf1/Cip1^ and MSI1 expression in the YFP^+^ cells following radiation-induced injury (Supplementary Fig. 5D). Previous studies showed that MSI1-ablated rISCs were blocked in G1 phase. Therefore, this finding suggests that the first step of rISC activation is MSI1/2 independent. However, MSI proteins are required to stimulate cell cycle progression. P21^Waf1/Cip1^ inhibits, among others, the G1/S transition^56^, and MSI1 is crucial for downregulating *p21*^*Waf1/Cip1*^ expression to promote the cell cycle. Our finding that increasing *Msi1* expression corresponded with decreasing *p21*^*Waf1/Cip1*^ expression and increasing EdU incorporation (indicating S phase) between 48 and 96 h post-injury suggests that a well-established mechanism of negative regulation of p21^Waf1/Cip1^ by MSI1 may occur in vivo. The exact mechanism of MSI1 activation is not fully understood. Over the years, numerous hypotheses, including β catenin-dependent mitotic spindle formation^36^ or WNT signaling activation, have been proposed^27,34,57–59^. However, most current findings indicate that MSI1 is likely activated through PTEN-PI3K-AKT signaling, which leads to mTORC1 activation downstream of MSI^28,31,60^.

Previously, we demonstrated that after γ irradiation-induced injury in vivo*,* KLF4 acts as a radioprotective factor by inhibiting apoptosis and contributing to crypt regeneration^32^. Interestingly, this phenomenon was correlated with increased proliferation, which showed that KLF4 function is context-dependent and may become pro-proliferative upon injury^16^. In the current study, we confirmed that regeneration driven by *Bmi1-Cre*^*ER*^ marked cells is reduced following KLF4 ablation, as shown by a reduced number of YFP^+^ cells, YFP^+^ cells incorporating EdU, and YFP^+^ surviving crypts during the regenerative phase in the *Bmi1*^*∆Klf4*^ mice compared to the *Bmi1*^*Ctrl*^ mice (Figs. 4 and 6 and Supplementary Figs. 7 and 10). We further characterized the context-dependent pro-proliferative function of KLF4 upon injury by showing that it is associated with maintaining *Msi1* expression (96 h post-injury). We showed that *Bmi1*-specific *Klf4* deletion resulted in a reduced number of YFP^+^MSI1^+^ cells (Fig. 5A) during the late postinjury phase. Furthermore, we identified several putative KLF4 binding sites in the *Msi1* promoter sequence and demonstrated that KLF4 directly binds to at least three of them in vitro using ChIP-PCR (Fig. 5I). A luciferase assay together with an in vitro study of the effect of KLF4 on MSI1 levels demonstrated that KLF4 positively regulates *Msi1* expression (Fig. 5B–H). In summary, our data confirm and constitute an important complement to the existing data regarding the nature of p21^Waf1/Cip1^ and MSI1 expression in radiation-injured *Bmi1-Cre*^*ER*^ cells. Additionally, we provide the first experimental proof of a new KLF4 target gene and explain the previously described pro-proliferative effect of KLF4 upon γ irradiation-induced gut injury. Moreover, we showed that irradiation of single sorted YFP^+^-derived organoids depicts an in vivo response to γ irradiation and that organoids grown from YFP^+^ cells isolated from the *Bmi1*^*ΔKlf4*^ mice are more radiosensitive than those isolated from the *Bmi1*^*Ctrl*^ mice.

## Materials and methods

### Mouse strains and treatment

*Bmi1-Cre*^*ER*^; *Rosa26*^*eYFP*^ (*Bmi1*^*Ctrl*^) mice and *Bmi1-Cre*^*ER*^; *Rosa26*^*eYFP*^*;* *Klf4*^*fl/fl*^ (*Bmi1*^*∆Klf4*^) mice were described previously^16,61^. The mice were given normal chow and water ad libitum. A routine PCR protocol was used for genotyping tail DNA samples. The primers used were as follows: Bmi1Cre-F (5′-ACCAGCAACAGCCCCAGTGC-3′), Bmi1Cre-R wt (5′-TAGGCATTAATTGAGATTAACAAACTA-3′), Bmi1Cre-R mut (5′-AAAGACCCCTAGGAATGCTC-3′), Rosa26eYFP-F (5′-AAAGTCGCTCTGAGTTGTTAT-3′), Rosa26eYFP-R wt (5′-GGAGCGGGAGAAATGGATATG-3′), Rosa26eYFP-R mut (5′-AAGACCGCGAAGAGTTTGTC-3′), Klf4-F (5′-CTGGGCCCCCACATTAATGAG-3′), and Klf4-R floxed (5′-CGCTGACAGCCATGTCAGACT-3′). The expected product sizes are as follows: Bmi1 wt, 421 bp; Bmi1-Cre, 365 bp; Rosa26 wt, 600 bp; Rosa26eYFP, 320 bp; Klf4 wt, 172 bp; and Klf4 floxed, 296 bp. Only 8- to 12-week-old and gender-matched mice were used in this study. Tamoxifen (Sigma-Aldrich) was dissolved in corn oil (30 mg/ml) and administered by a single intraperitoneal injection (225 mg/kg) according to the experimental design described in Supplementary Fig. 1. The experimental group was then exposed to γ-irradiation (^137^Cs) at a dose rate of 0.8 Gy/min for a total of 12 Gy TBI, whereas the sham group was injected with tamoxifen and received 0 Gy TBI. The mice were euthanized, and the proximal part of the small intestine (PSI) was collected at 0, 6, 24, 48, 72 and 96 h (protocol 1, Supplementary Fig. 1A) or at 48, 72 and 96 h (protocol 2, Supplementary Fig. 1B) from the time of irradiation and formalin fixation. Three hours prior to euthanasia, all mice were injected with 100 μg of EdU dissolved in 1:5 DMSO and water. All studies and procedures involving animal subjects were approved by the Stony Brook University Institutional Animal Care and Use Committee (IACUC) and conducted strictly in accordance with the approved animal handling protocol.

### IF staining

PSIs were swiss-rolled as described previously^16^, and paraffin-embedded blocks were cut into 4 μm tick sections. The tissues were deparaffinized in xylene, incubated at room temperature in 2% hydrogen peroxide in methanol for 30 min, rehydrated in an ethanol gradient and incubated in a 10 mM Na-citrate buffer (pH 6.0) at 120 °C for 10 min in a pressure cooker to retrieve antigens. The sections were then washed with water, incubated for 1 h at 37 °C in a blocking solution (5% BSA and 0.01% Tween 20 in 1 × Tris-based PBS), and incubated with primary antibodies against GFP (1:500, AvesLabs), p21^Waf1/Cip1^ (1:200, BD Bioscience), KLF4 (1:300, R&D Systems), and MSI1 (1:200, MBL International Corporation) overnight at 4 °C. EdU-labeled cells were stained using the Click-IT plus EdU imaging kit (Thermo Fisher) according to the manufacturer’s instructions. The tissues were also counterstained with Hoechst 33258 to visualize the nuclei. Microscopic images were obtained using an Eclipse 90i fluorescence microscope (Nikon) equipped with a DS-Qi1Mc camera (Nikon).

### Cell and crypt scoring

At least 20 YFP positive (YFP^+^) half-crypts were selected for counting from each mouse (*n* = 3). The numbers are represented as the average percent of stained cells of the total number of cells in the YFP^+^ crypt and as a percent of the costained YFP^+^ cells of all YFP^+^ cells within a crypt ± SD.

### YFP^+^ cell isolation for total RNA analysis

Mice were treated according to protocol 1 presented in Supplementary Fig. 1A. Proximal small intestines were harvested from both the sham and irradiated mice at 0, 6, 24, 48, 72 and 96 h. Duodena were flushed using ice-cold PBS and cut open longitudinally, and villi were scraped using a glass slide. Tissues were washed in PBS and incubated for 45 min in PBS containing 5 mM EDTA at 4 °C with shaking. Then, the tissues were vigorously shaken manually for 30 s, and the suspension was transferred to a new tube and harvested by centrifugation at 800 rpm for 5 min at 4 °C. The pellet was resuspended in 4 × TrypLE (Gibco) prewarmed to 37 °C and incubated with rotation for 50 min at 37 °C with occasional manual shaking. DMEM was added to dilute 4 × TrypLE to 2×, and the cells were centrifuged at 900 rpm at 4 °C for 5 min. The supernatant was removed, and isolated cells were resuspended in DMEM and sorted by flow cytometry (BD FACSAria III). At least 1 × 10^5^ YFP^+^ FACS-sorted cells per sample were used for total RNA isolation. During the entire procedure, aluminum foil was used to wrap the Falcon tube and preserve fluorescence.

### RNA isolation and gene expression analysis of cells by qRT-PCR

Total RNA was isolated using an RNeasy Mini Kit (Qiagen), and an RNase-Free DNase Set (Qiagen) was used to remove genomic DNA. The RNA was examined for purity and concentration using a NanoDrop spectrophotometer (NanoVue Plus, GE Healthcare). First strand complementary DNA (cDNA) was synthesized using 2 µg of total RNA and the SuperScript VILO cDNA Synthesis Kit (Thermo Fisher). The reaction was performed using a standard protocol for 10 min at 25 °C followed by 10 min at 50 °C and 5 min at 85 °C in a Mastercycler X50s system (Eppendorf). qRT-PCR analysis was performed using TaqMan Gene Expression Master Mix (Thermo Fisher) and QuantStudio 3 (Applied Biosystems) with 10 min at 95 °C followed by 40 cycles of 15 s at 95 °C and 1 min at 60 °C. Commercially available TaqMan primers detecting mouse *Cdkn1a* (Mm00432448-FAM), *Msi1* (Mm01203522-FAM), *Klf4* (Mm00516104-FAM), and *Hprt1* (Mm03024075-VIC) and human *CDKN1A* (Hs00355782-FAM), *MSI1* (Hs01045894-FAM), *KLF4* (Hs00358836-FAM), and *HPRT1* (Hs02800695-VIC) transcripts were used. All kits were used according to the manufacturer’s instructions.

### Cell culture and in vitro assays

The human embryonic kidney HEK293T cell line was purchased from the American Type Culture Collection (CRL-3216) and maintained in Dulbecco’s modified Eagle’s medium (DMEM) supplemented with 10% fetal bovine serum (FBS) and 1% penicillin/streptomycin (P/S) at 37 °C in a 5% CO_2_ incubator. Additionally, a gene encoding *Gfp* was permanently introduced into the genome of HEK293T cells using the lentiviral system as described previously (HEK293T-GFP)^62,63^. For in vitro analysis of the effect of MSI1 on endogenous *CDKN1A* expression, cells were γ-irradiated with a 12 Gy dose and 24 h later transfected with the pCMV6-AC-GFP-*MSI1* plasmid (OriGene) encoding human *MSI1* or pcDNA3.1 as an empty vector control using Lipofectamine 2000 (Thermo Fisher) in Opti-MEM medium (Gibco). Both floating and adherent cells were collected at 0, 24, 48 and 72 h after irradiation. We calculated the ratios of mRNA or protein levels after irradiation vs. with the sham treatment for samples transfected with either the empty vector control or *MSI1*-overexpressing vector. For in vitro analysis of the effect of KLF4 on endogenous *MSI1* expression, cells were transfected with the pcDNA3.1-KLF4 (FL) plasmid coding human *KLF4*^64^ or pcDNA3.1 as an empty vector control using Lipofectamine 2000 (Thermo Fisher) in Opti-MEM medium (Gibco). The cells were collected at 0, 24, 48 and 72 h post-transfection. qRT-PCR analysis of gene expression and Western blot analysis of protein levels were performed. Each experiment was performed in triplicate.

### Western blot analysis

Both floating and adherent cells were collected, washed with cold PBS and lysed in 1 × Laemmli buffer. Proteins were electrophoresed on 4–20% polyacrylamide gels (Bio-Rad) and transferred to nitrocellulose membranes (Bio-Rad). The membranes were blocked with 5% nonfat milk in 1 × TBS containing 0.01% Tween 20 for 1 h at room temperature and incubated on a rocking platform overnight with primary antibodies against p21^Waf1/Cip1^ (1:2500, BD Pharmingen), MSI1 (1:2000, MBL International Corporation), KLF4 (1:2000, MBL International Corporation), GAPDH (1:5000, Millipore) and α-tubulin (1:5000, Abcam). The membranes were then washed, incubated with appropriate IgG secondary antibodies conjugated with HRP for 1 h at room temperature and developed using SuperSignal West Pico PLUS Chemiluminescent Substrate (Thermo Fisher) and X-ray films. The membranes were probed for α-tubulin or GAPDH as an internal control. Loading controls were run on the same blots. Relative band density was quantified using ImageJ 1.8.0_112 (National Institute of Health, Bethesda, MD) software.

### Luciferase assay

HEK293T-GFP cells were seeded in 96-well plates at 1 × 10^4^ cells per well. GFP expression was used as an endogenous control. Day later, the cells were cotransfected using Lipofectamine 2000 (Invitrogen) and Opti-MEM medium (Gibco). The effect of mouse MSI1 on *Cdkn1a* expression was tested using 100 ng/well of the pGL3-Basic vector (Addgene) as an empty vector control and 100 ng/well of pGL3-p21^Waf1/Cip1^ 3′UTR (Addgene) encoding the 3′UTR sequence of the mouse *Cdkn1a* gene and *luc* encoding luciferase from *Photinus pyralis*. Mouse *Msi1* was overexpressed using 100 ng/well of the pCDH-CMV-Msi1 (FL) vector (Addgene), and 100 ng/well of pReceiver-Lv216 (GeneCopoeia) was used as an empty vector control. Luciferase activity was measured 48 h post-transfection using a Dual-Luciferase Reporter Assay (Promega) according to the manufacturer’s instructions. Luciferase activity was calculated as a ratio of the signal obtained from cells transfected with the pGL3-*P21-3′UTR* vector *vs*. the pGL3-Basic vector.

The effect of mouse KLF4 on *Msi1* expression was tested using 100 ng/well of the pEZX-PG02 vector (GeneCopoeia) encoding the mouse *Msi1* promoter sequence and *Gaussia* luciferase. One hundred nanograms/well of the pcDNA3.1 vector (Addgene) was used as an empty vector control, and *Klf4* was overexpressed using the pcDNA3.1-KLF4 FL vector for mouse full-length *Klf4* or pcDNA3.1-KLF4 ∆ZFD for the mouse *Klf4* mutant with deletion of the C-terminal DNA-binding domains and encoding amino acids 1–349 of the full-length protein^64^. Luciferase activity was measured 72 h post-transfection using the Secrete-Pair Dual Luminescence Assay Kit (GeneCopoeia) according to the manufacturer’s instructions and calculated as a ratio of the signal obtained from cells transfected with vector encoding *Klf4 *vs. the empty vector.

### ChIP-PCR analysis

Approximately 3 × 10^6^ HEK293T-GFP cells were seeded on a 100 mm dish, and 24 h later, the cells were transfected with 500 ng of pEZX-PG02 (GeneCopoeia) encoding the mouse *Msi1* promoter sequence and 500 ng of the pcDNA3.1.-KLF4(FL) vector encoding full-length mouse *Klf4*^64^ using Lipofectamine 2000 (Invitrogen). The cells were collected 24 h post-transfection, and ChIP was performed using a Simple ChIP kit with magnetic beads (Cell Signaling) according to the manufacturer’s instructions. After DNA was digested with nuclease, it was sonicated to an average length of 100–500 bp fragments through three pulses, each 10 s long, at 25% of the maximum power using an ultrasonic liquid processor (Q800R System, QSonica). For KLF4 immunoprecipitation, 10 or 15 µl of rabbit antibodies against KLF4 was used (MBL International Corporation). Normal rabbit IgG antibodies were used as a negative control, and rabbit histone H3 IgG antibodies were used as a positive control. The mouse *Msi1* promoter sequence was obtained from the Eukaryotic Promoter Database (Ref sequence NM_002442), and using the JASPAR algorithm, we identified putative KLF4 binding sites up to − 1.5 kb within the proximal promoter. The primers used for the PCR analysis were designed using Primer3 and the IDT PrimerQuest tool. The following primers were used: 1F (5′-GCTAAAGAGCCAGGAGTTAGAG-3′), 1R (5′-GCCCTTGCTGTCCAAATTAAG-3′), 2F (5′-CTTACCAGTTGGAAGGTGTTGG-3′), 2R (5′-GACGGACAGGCCATGCT-3′), 3F (5′-GGAGGTGACAACTTGGGAAA-3′), 3R (5′-CCAACTGGTAAGAAACCTCTCC-3′), 4F (5′-CTACCTTGAACGCACCGGGA-3′), and 4R (5′-CTCGGGGTTCCTGTGTGTCC-3′). The expected product sizes are as follows: 1 set: 445 bp, 2 set: 482 bp, 3 set: 347 bp, and 4 set: 161 bp. PCR was performed using PCR Taq Blue Master Mix (Thermo Fisher) and a standard protocol: a 5 min initial denaturation at 95 °C, 34 cycles of 30 s at 95 °C, 30 s at 62 °C and 30 s at 72 °C and a 5 min final elongation at 72 °C. For each reaction, 1 μl of isolated and purified DNA was used. The experiment was performed in triplicate.

### YFP^+^ cell isolation for organoid culture and assessment of regeneration

Proximal small intestines were harvested from both the *Bmi1*^*Ctrl*^ and *Bmi1*^*ΔKlf4*^ mice 48 h after tamoxifen injection. Intestinal epithelial cells were dissociated as previously described^65^. Single YFP^+^ cells were sorted by flow cytometry (BD FACSAria III), and 2000 cells were embedded in Matrigel (Corning, Corning, NY) and dispensed into 24-well plates as 25 μl droplets. Organoid culture medium was prepared using the L-WRN cell line as previously described^66^ and supplemented with 1 × N2 supplement (Thermo Fisher Scientific), 1 × B27 supplement (Thermo Fisher), 10 nM gastrin I (Sigma-Aldrich), 50 ng/ml recombinant human epidermal growth factor (Thermo Fisher), 500 nM transforming growth factor β inhibitor A83-01 (Tocris Bioscience, Bristol, United Kingdom), 1 mM *N*-acetylcysteine (Sigma-Aldrich) and 100 μg/ml Primocin antibiotic cocktail (Thermo Fisher). The GSK3β inhibitor CHIR99021 (10 μM) (Tocris) and ROCK inhibitor Y-27632 (10 μM) (Sigma-Aldrich) were also added during the first 2 days of culture. The media were changed every 2 days. Live organoids were imaged using an Eclipse Ti2 inverted microscope (Nikon) equipped with a DS-Qi2 camera (Nikon). After 4 days of culture, organoids were exposed to 0, 6, 8, 10 or 12 Gy of γ irradiation (^137^Cs) and continued to culture for an additional 7 days. Organoid numbers were calculated by manual counting under a bright field microscope at days 0 and 7 postirradiation. The regeneration rate was calculated as the number of organoids 7 days postirradiation *vs*. the number of organoids at 0 h.

### YFP^+^ crypt survival

To compare YFP^+^ crypt survival, we quantified actively proliferating YFP^+^ crypts at 72 h and 96 h post-injury in the sham or irradiated *Bmi1*^*Ctrl*^ and *Bmi1*^*ΔKlf4*^ mice treated according to protocol 1 (Supplementary Fig. 1A). We used IF-stained tissues for YFP and EdU analysis. We considered crypts to be actively proliferating if they contained at least three YFP^+^ cells that were costained with EdU, indicating S-phase of the cell cycle. For each tissue specimen, we used the same length consisting of 200 crypts. First, we calculated the ratio of YFP^+^ proliferating crypts *vs*. all YFP^+^ crypts in designated fragments of small intestine specimens for the sham or irradiated samples. Next, we calculated the ratio of actively proliferating YFP^+^ crypts after irradiation vs. after sham treatment for certain time points. The experiment was performed in triplicate. The data are represented as the average percent of surviving YFP^+^ crypts ± SD.

### Statistical analysis

Statistical analyses were performed with GraphPad Prism version 8.00 for Windows (GraphPad Software, San Diego, CA). One-way ANOVA, Student’s t-test or correlation analysis was used depending on the purpose. Data are expressed as the mean ± SD. All experiments were performed in at least three independent replicates. p-values < 0.05 were considered statistically significant. Post hoc analysis of normal distribution was performed whenever applicable.

### Consent for publication

All authors agree to the publication of this manuscript.

### Ethics approval and consent to participate

All studies and procedures involving animal subjects were approved by the Stony Brook University Institutional Animal Care and Use Committee (IACUC) and conducted strictly in accordance with the approved animal handling protocol.

## Supplementary information

Supplementary Figures.

## Data Availability

All data analyzed in this study are presented in this article and available upon request. Correspondence and requests for materials should be addressed to A.B.B.
