# Supplementary material for: Interplay among p21Waf1/Cip1, MUSASHI-1 and Krüppel-like factor 4 in activation of Bmi1-CreER reserve intestinal stem cells after gamma radiation-induced injury
Source: Sci Rep. 2020 Oct 27;10:18300. doi: 10.1038/s41598-020-75171-w (PMC7591575; doi:10.1038/s41598-020-75171-w)
Supplement: Supplementary file 1 — Supplementary Figures. [file 41598_2020_75171_MOESM1_ESM.pdf]

Supplementary Information

Interplay among p21$^{Waf1/Cip1}$, MUSASHI-1 and Krüppel-like factor 4 in activation of *Bmi1*-CreER reserve intestinal stem cells after gamma radiation-induced injury

Emilia J. Orzechowska[1,2], Takahito Katano[1,3], Agnieszka B. Bialkowska[1*], Vincent W. Yang[1,4,*]

[1] Department of Medicine, Renaissance School of Medicine at Stony Brook University, Stony Brook, New York, USA

[2] Department of Molecular Biology, Faculty of Biology, University of Warsaw, Warsaw, Poland

[3] Department of Gastroenterology and Metabolism, Nagoya City University Graduate School of Medical Sciences, Nagoya, Japan

[4] Department of Physiology and Biophysics, Renaissance School of Medicine at Stony Brook University, Stony Brook, New York, USA

[*] Electronic address: Vincent.Yang@stonybrookmedicine.edu and Agnieszka.Bialkowska@stonybrookmedicine.edu

Supplementary Figure 1

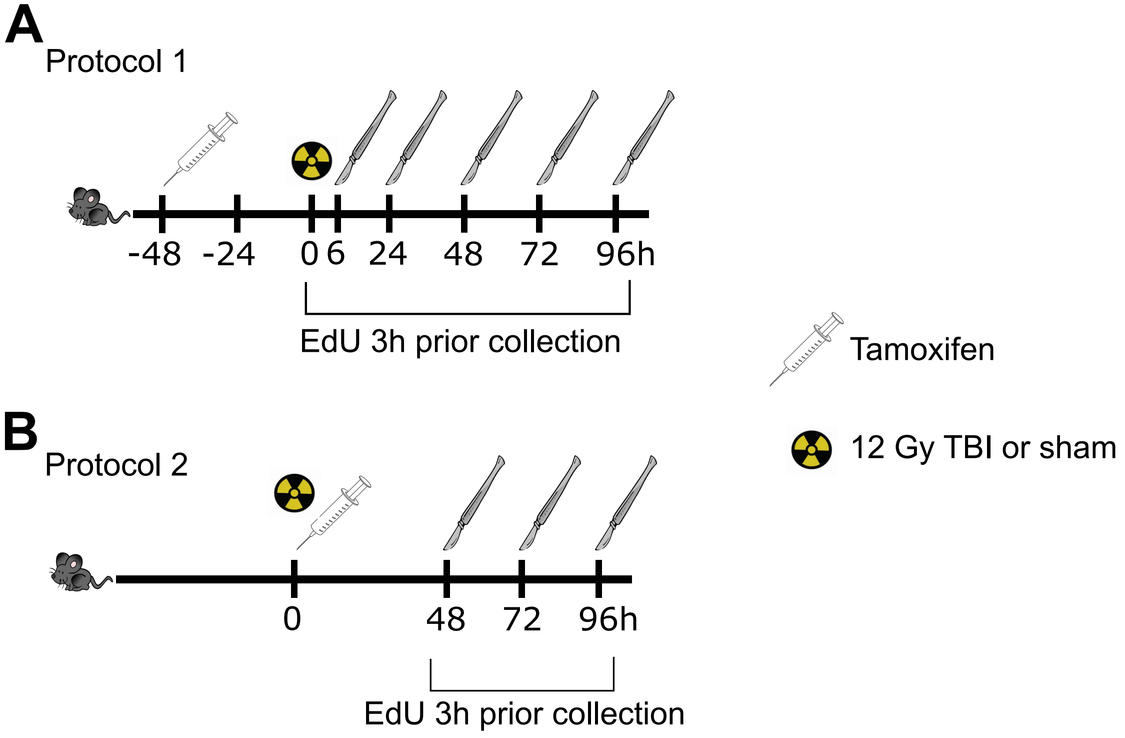

Supplementary Figure 1. Outline of the experimental design. (A) Eight-to 12-week-old female *Bmi1*^{Ctrl} and *Bmi1*$^{\Delta Klf4}$ mice were injected with tamoxifen 2 days prior treatment and sacrificed at 0, 6, 24, 48, 72 or 96 h after irradiation with total dose of 0 Gy (sham group) or 12 Gy (irradiated group) total body irradiation. Mice were injected with EdU 3 h prior to sacrifice. (B) Eight-to 12-week-old female *Bmi1*^{Ctrl} and *Bmi1*$^{\Delta Klf4}$ mice were injected with tamoxifen, irradiated at time 0 h with total dose of 0 Gy (sham group) or 12 Gy (irradiated group) total body irradiation and sacrificed at 48, 72 or 96 h after irradiation. Mice were injected with EdU 3 h prior to sacrifice

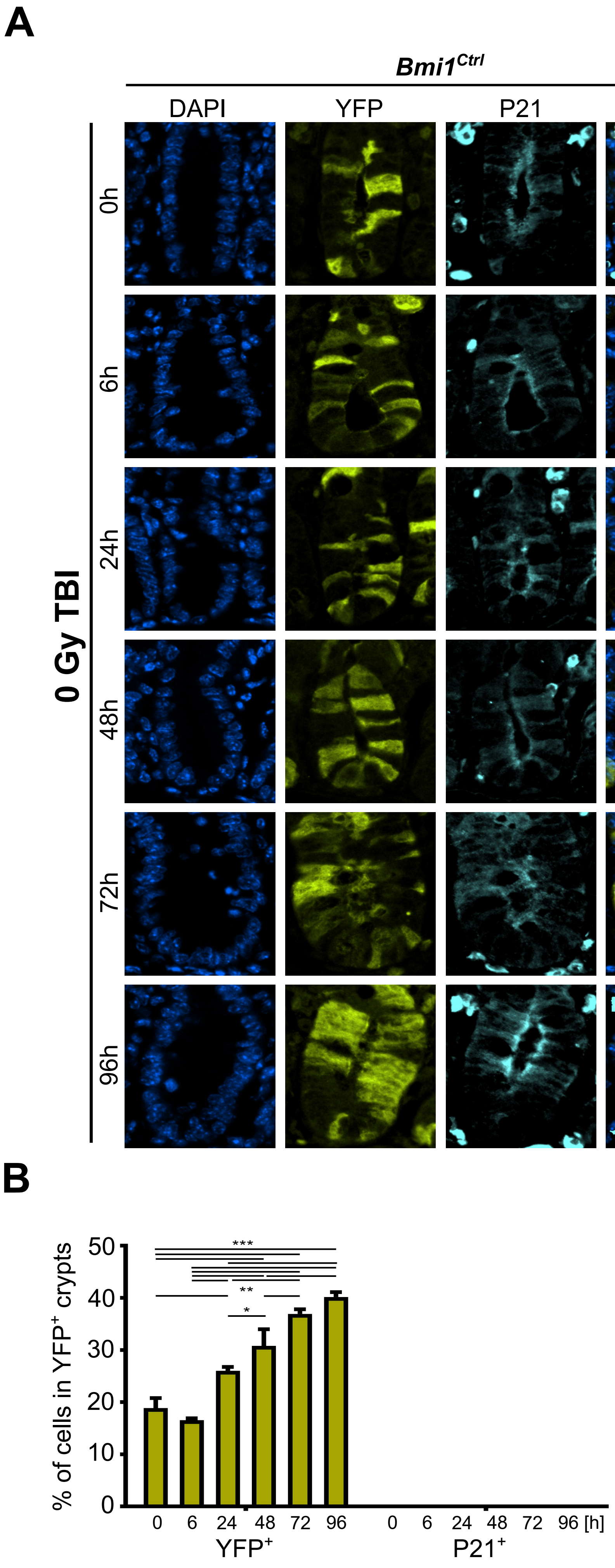

Supplementary Figure 2. Time-dependent p21$^{Waf1/Cip1}$ (P21) expression pattern in the YFP^{+} crypts after 0 Gy TBI of the *Bmi1^{Ctrl}* mice treated according to protocol 1 (Supplementary Fig. 1A). (A) Representative IF images of DAPI, YFP, and p21$^{Waf1/Cip1}$ staining in the PSI crypts at 0, 6, 24, 48, 72 and 96 h obtained under a fluorescence microscope. The scale bar represents 20 µm. (B) Quantification of the percentage of YFP^{+} or p21$^{Waf1/Cip1}$ $^{+}$ cells in the YFP^{+} crypts. Data are represented as the mean ± SD, 20 YFP^{+} crypts were quantified per mouse, and n = 3 mice per group. * $p < 0.05$, ** $p < 0.01$ and *** $p < 0.001$ by one-way ANOVA.

Supplementary Figure 3

A

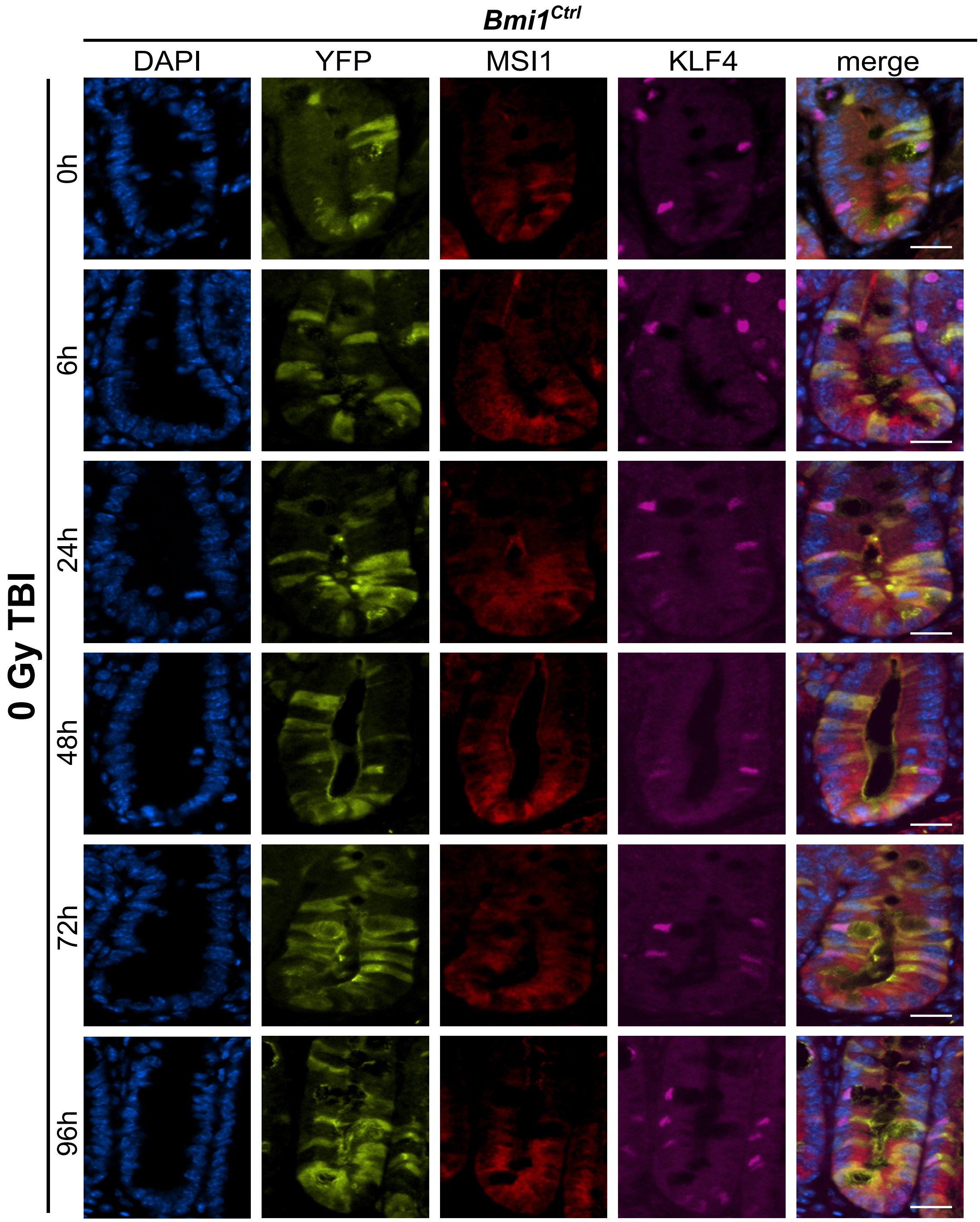

B

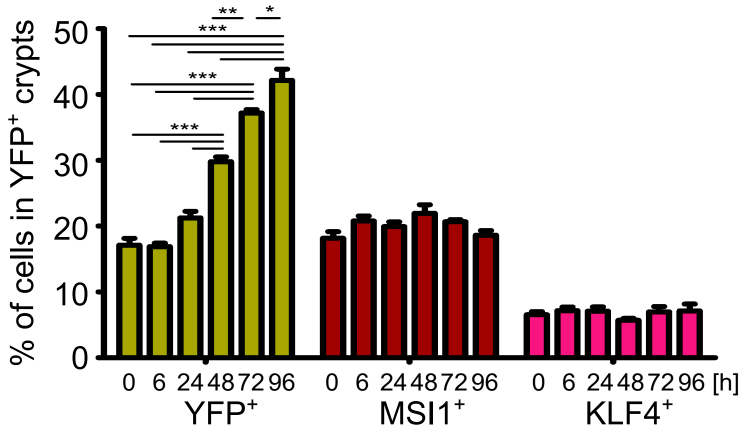

C

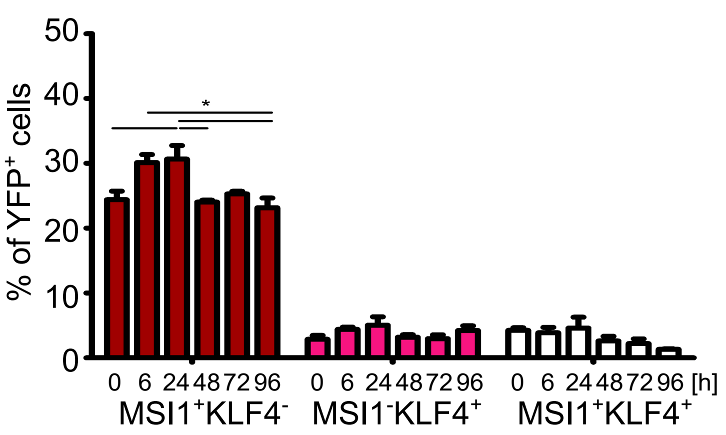

Supplementary Figure 3. Time-dependent MSI1 and KLF4 expression patterns in the YFP+ crypts after 0 Gy TBI of the *Bmi1*Ctrl mice treated according to protocol 1 (Supplementary Fig. 1A). (A) Representative IF images of DAPI, YFP, MSI1 and KLF4 staining in the PSI crypts at 0, 6, 24, 48, 72 and 96 h obtained under a fluorescence microscope. The scale bar represents 20 μm. (B) Quantification of the percentage of YFP+ or MSI1+ or KLF4+ cells in the YFP+ crypts. (C) Quantification of the percentage of YFP+ cells co-stained with MSI1, KLF4 or MSI1 and KLF4 together. Data are represented as the mean ± SD, 20 YFP+ crypts were quantified per mouse, and n = 3 mice per group.
* $p < 0.05$, ** $p < 0.01$ and *** $p < 0.001$ by one-way ANOVA.

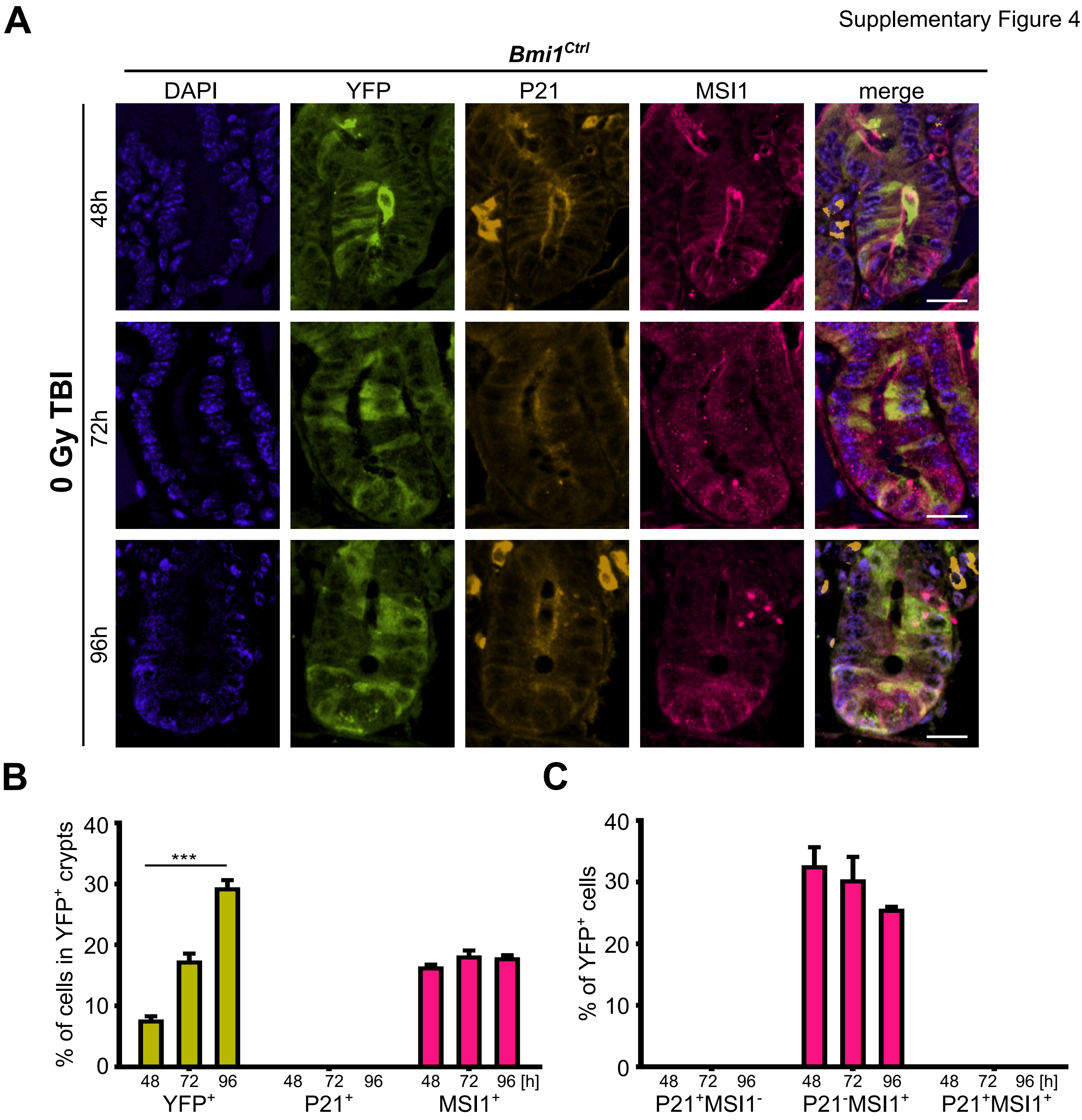

Supplementary Figure 4. Time-dependent $p21^{Waf1/Cip1}$ (P21) and MSI1 co-expression patterns in YFP^+ crypts after 0 Gy TBI of the *Bmi1*^{Ctrl} mice treated according to protocol 2 (Supplementary Fig. 1B). (A) Representative IF images of DAPI, YFP, $p21^{Waf1/Cip1}$, and MSI1 in the PSI crypts at 48, 72 and 96 h after tamoxifen injection obtained under a fluorescence microscope. The scale bar represents 20 µm. (B) Quantification of the percentage of YFP^+, $p21^{Waf1/Cip1+}$ or $MSI1^+$ cells in the YFP^+ crypts. (C) Quantification of the percentage of YFP^+ cells costained with $p21^{Waf1/Cip1}$, MSI1 or $p21^{Waf1/Cip1}$ and MSI1 together. Data are represented as the mean ± SD, 20 YFP^+ crypts were quantified per mouse, and n = 3 mice per group. *** $p < 0.001$ by one-way ANOVA.

Supplementary Figure 5

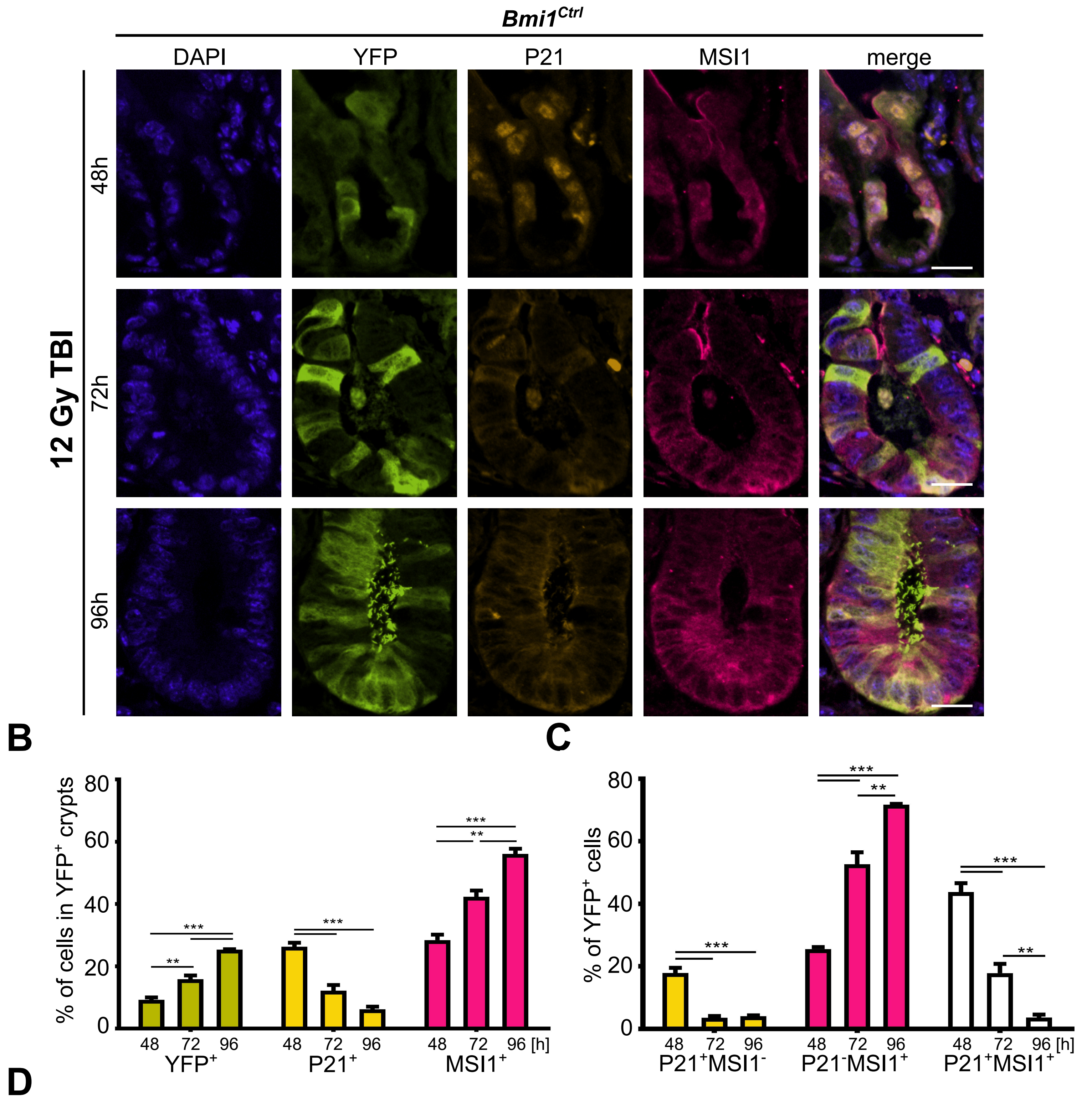

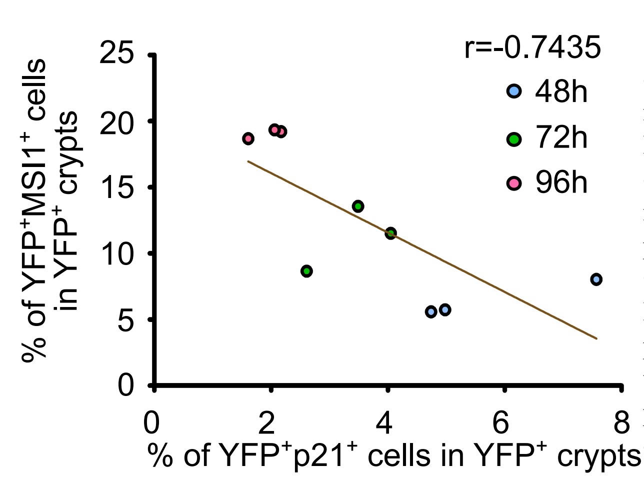

Supplementary Figure 5. Time-dependent p21$^{Waf1/Cip1}$ (P21) and MSI1 coexpression patterns in the YFP^{+} crypts after 12 Gy TBI of the *Bmi1^{Ctrl}* mice treated according to protocol 2 (Supplementary Fig. 1B). (A) Representative IF images of DAPI, YFP, p21$^{Waf1/Cip1}$, and MSI1 in the PSI crypts at 48, 72 and 96 h after irradiation obtained under a fluorescence microscope. The scale bar represents 20 μm. (B) Quantification of the percentage of YFP^{+}, p21$^{Waf1/Cip1+}$ or MSI1^{+} cells in the YFP^{+} crypts. (C) Quantification of the percentage of YFP^{+} cells costained with p21$^{Waf1/Cip1}$, MSI1 or p21$^{Waf1/Cip1}$ and MSI1 together. (D) Analysis of correlation between p21$^{Waf1/Cip1}$and MSI1 expression in the YFP^{+} crypts. Data are represented as the mean ± SD, 20 YFP^{+} crypts were quantified per mouse, and n = 3 mice per group. ** $p < 0.01$ and *** $p < 0.001$ by one-way ANOVA. Analysis of correlation by Spearman correlation test.

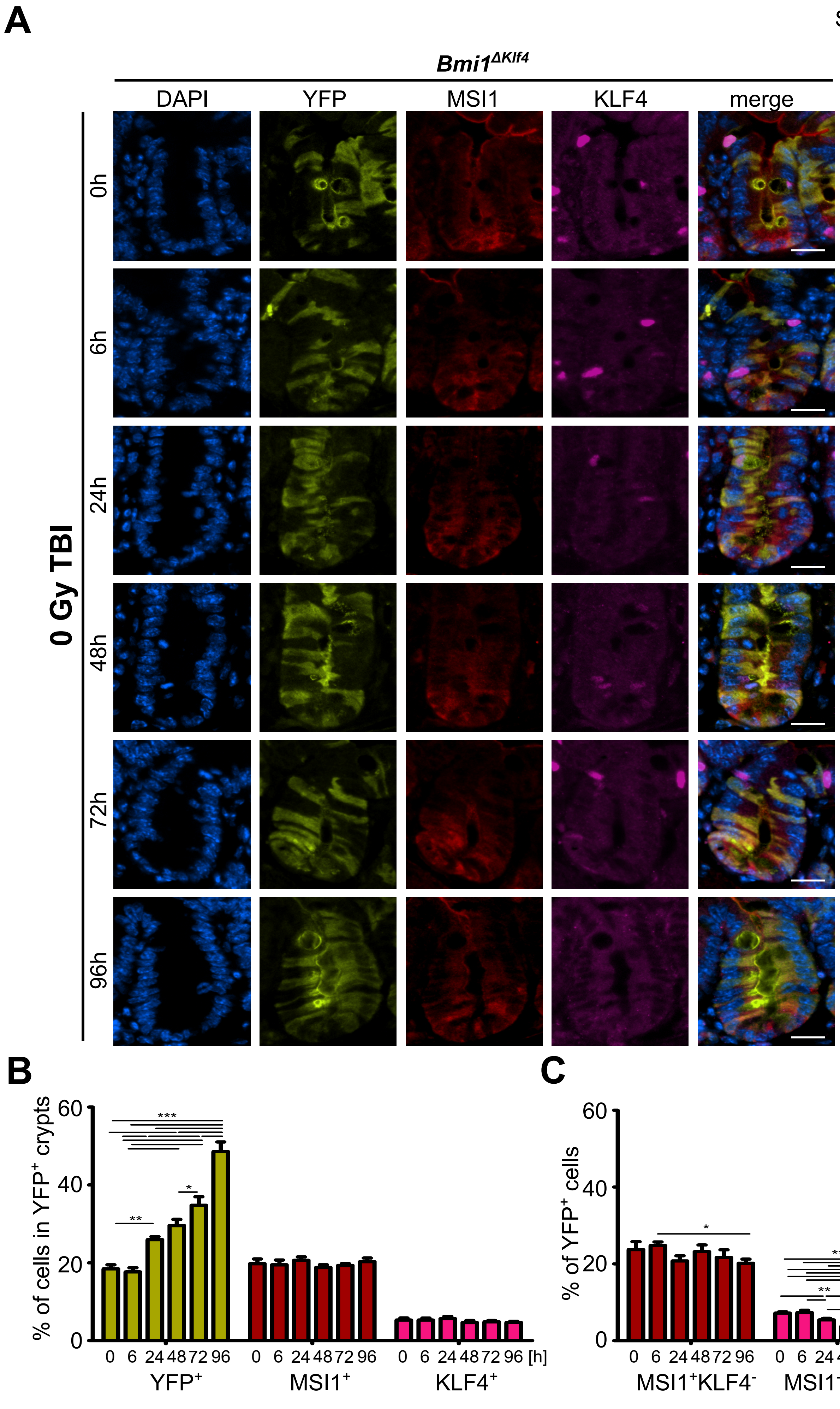

Supplementary Figure 6. Time-dependent MSI1 and KLF4 expression patterns in the YFP^{+} crypts after 0 Gy TBI of the *Bmi1*$^{\Delta Klf4}$mice treated according to protocol 1 (Supplementary Fig. 1A). (A) Representative IF images of DAPI, YFP, MSI1, and KLF4 in the PSI crypts at 0, 6, 24, 48, 72 and 96 h after irradiation obtained under a fluorescence microscope. The scale bar represents 20 μm. (B) Quantification of the percentage of YFP^{+}, MSI1^{+} or KLF4^{+} cells in the YFP^{+} crypts. (C) Quantification of the percentage of YFP^{+} cells costained with MSI1, KLF4 or MSI1 and KLF4 together. Data are represented as the mean ± SD, 20 YFP^{+} crypts were quantified per mouse, and n = 3 mice per group.
* $p < 0.05$, ** $p < 0.01$ and *** $p < 0.001$ by one-way ANOVA.

Supplementary Figure 7

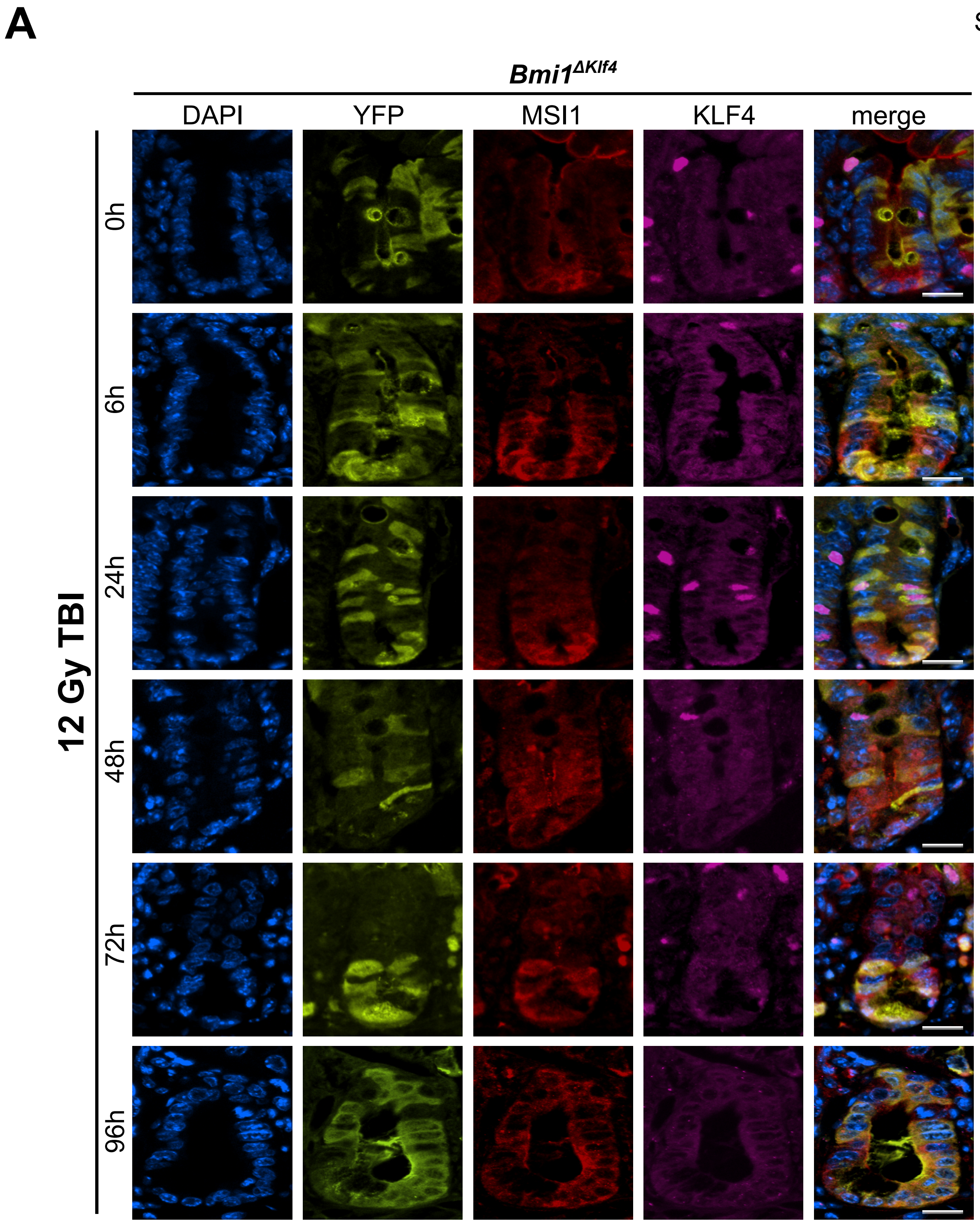

Supplementary Figure 7. Time-dependent MSI1 and KLF4 expression patterns in the YFP$^+$ crypts after 12 Gy TBI of the *Bmi1*$^{\Delta Klf4}$mice treated according to protocol 1 (Supplementary Fig. 1A). (A) Representative IF images of DAPI, YFP, MSI1, and KLF4 in the PSI crypts at 0, 6, 24, 48, 72 and 96 h after irradiation obtained under a fluorescence microscope. The scale bar represents 20 µm. (B) Quantification of the percentage of YFP$^+$, MSI1$^+$ or KLF4$^+$ cells in the YFP$^+$ crypts. (C) Quantification of the percentage of YFP$^+$ cells costained with MSI1, KLF4 or MSI1 and KLF4 together. Data are represented as the mean ± SD, 20 YFP$^+$ crypts were quantified per mouse, and n = 3 mice per group. * $p < 0.05$, ** $p < 0.01$ and *** $p < 0.001$ by one-way ANOVA.

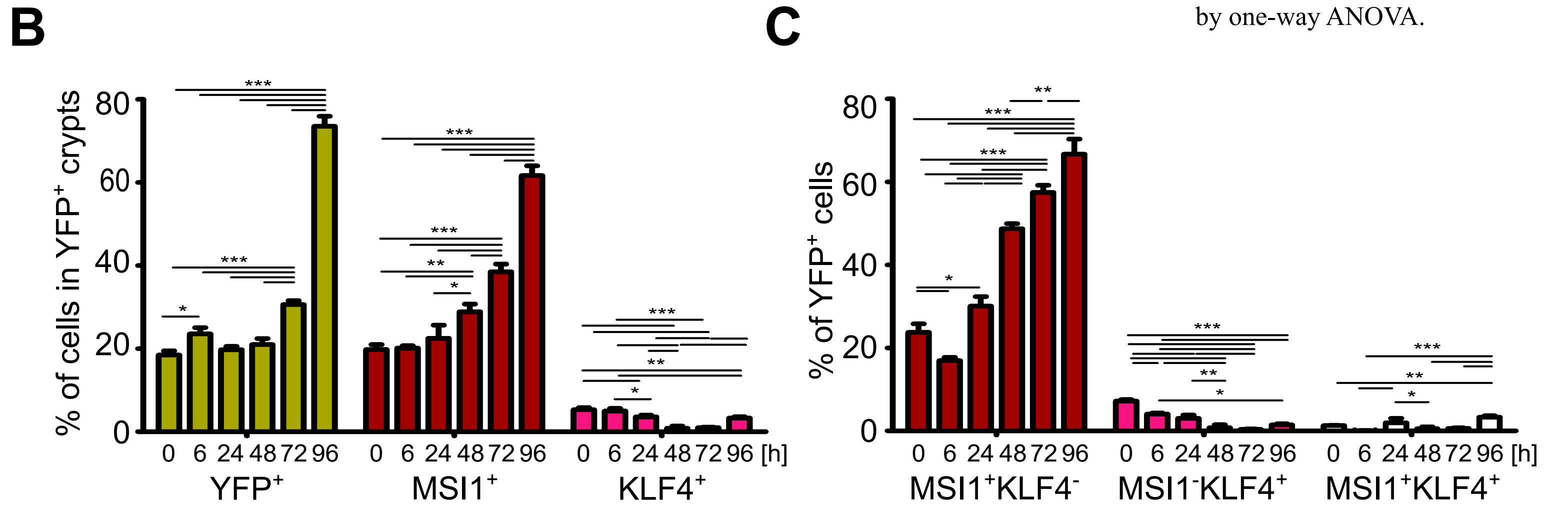

A

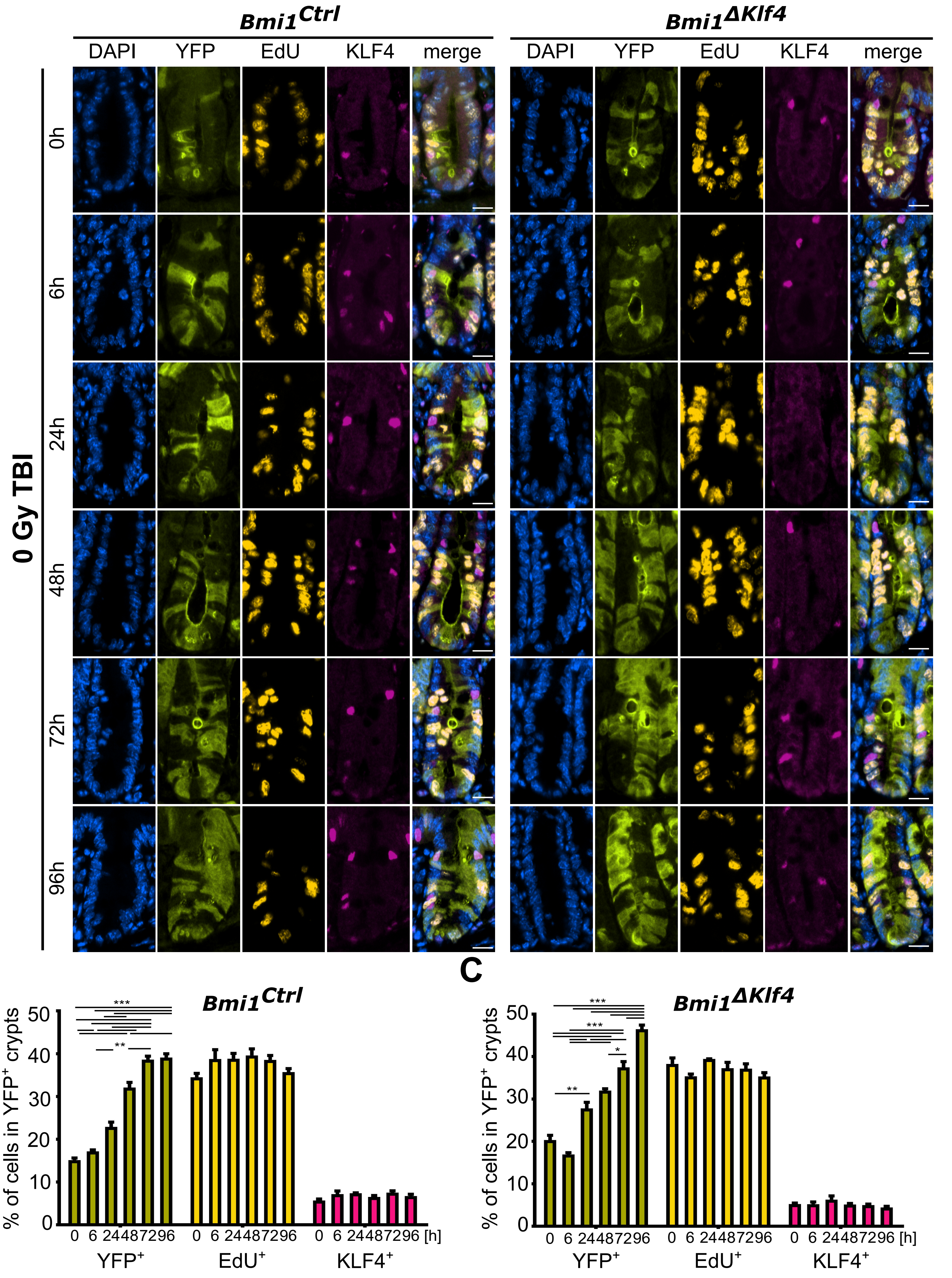
Bmi1Ctrl
Bmi1ΔKlf4
DAPI
YFP
EdU
KLF4
merge
0 Gy TBI
0h
6h
24h
48h
72h
96h
% of cells in YFP+ crypts
0
10
20
30
40
50
***
**
*
0 6 24 48 72 96
[h]
YFP+
EdU+
KLF4+

B

C

Supplementary Figure 8

D

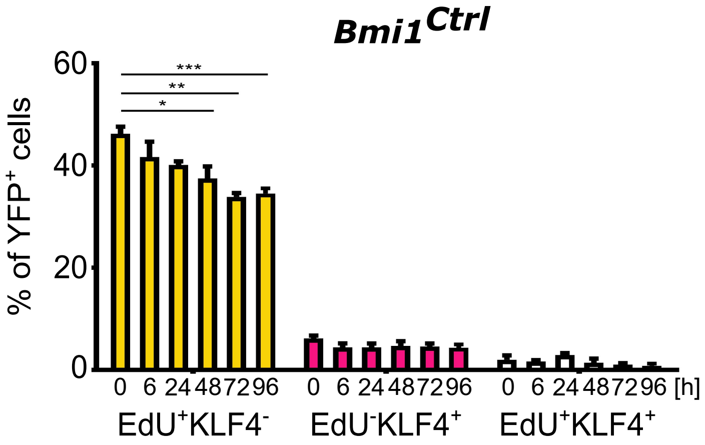

E

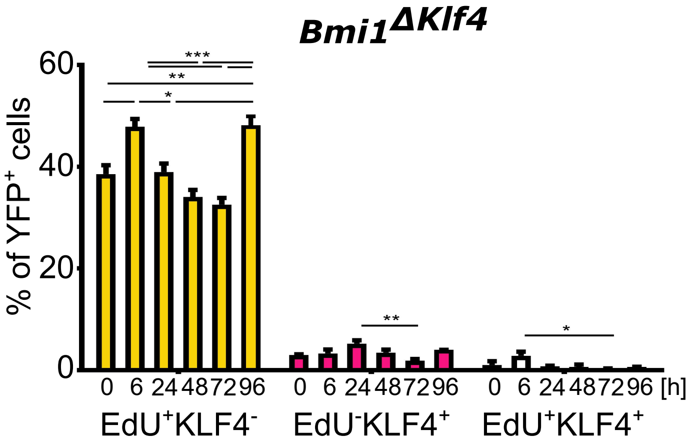

F

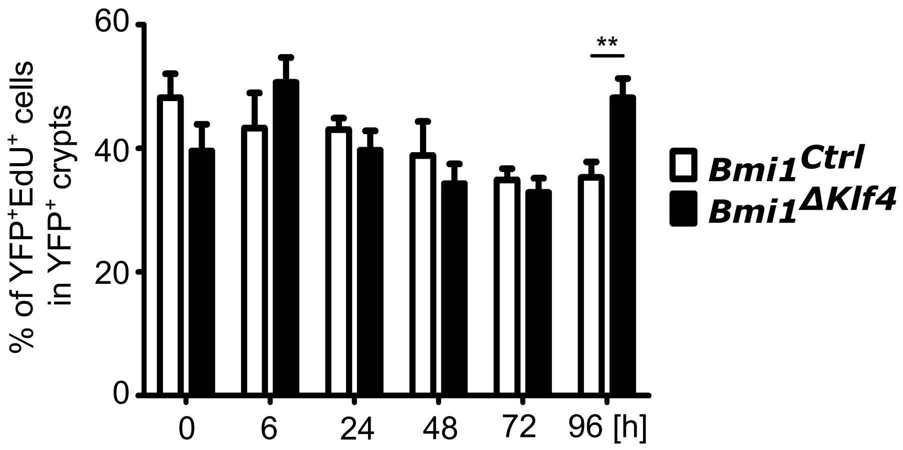

Supplementary Figure 8. KLF4 influences the proliferative ability of cells in the YFP$^+$ crypts after 0 Gy TBI of the *Bmi1*^{Ctrl}and *Bmi1*$^{\Delta Klf4}$mice treated according to protocol 1 (Supplementary Fig. 1A). (A) Representative IF images of DAPI, YFP, EdU, and KLF4 in the PSI crypts at 0, 6, 24, 48, 72 and 96 h obtained under a fluorescence microscope. The scale bar represents 20 µm. (B-C) Quantification of the percentage of YFP$^+$, EdU$^+$ or KLF4$^+$ cells in the YFP$^+$ crypts of the *Bmi1*^{Ctrl} (B) and *Bmi1*$^{\Delta Klf4}$(C) mice. (D-E) Quantification of the percentage of YFP$^+$ cells costained with EdU, KLF4 or EdU and KLF4 together of the *Bmi1*^{Ctrl}(D) and *Bmi1*$^{\Delta Klf4}$ (E) mice. (F) Comparison of the percentage of YFP$^+$EdU$^+$ cells in the YFP$^+$ crypts of the *Bmi1*^{Ctrl} and *Bmi1*$^{\Delta Klf4}$ mice. Data are represented as the mean ± SD, 20 YFP$^+$ crypts were quantified per mouse, and n = 3 mice per group. * $p < 0.05$, ** $p < 0.01$ and *** $p < 0.001$ by one-way ANOVA.

Supplementary Figure 9

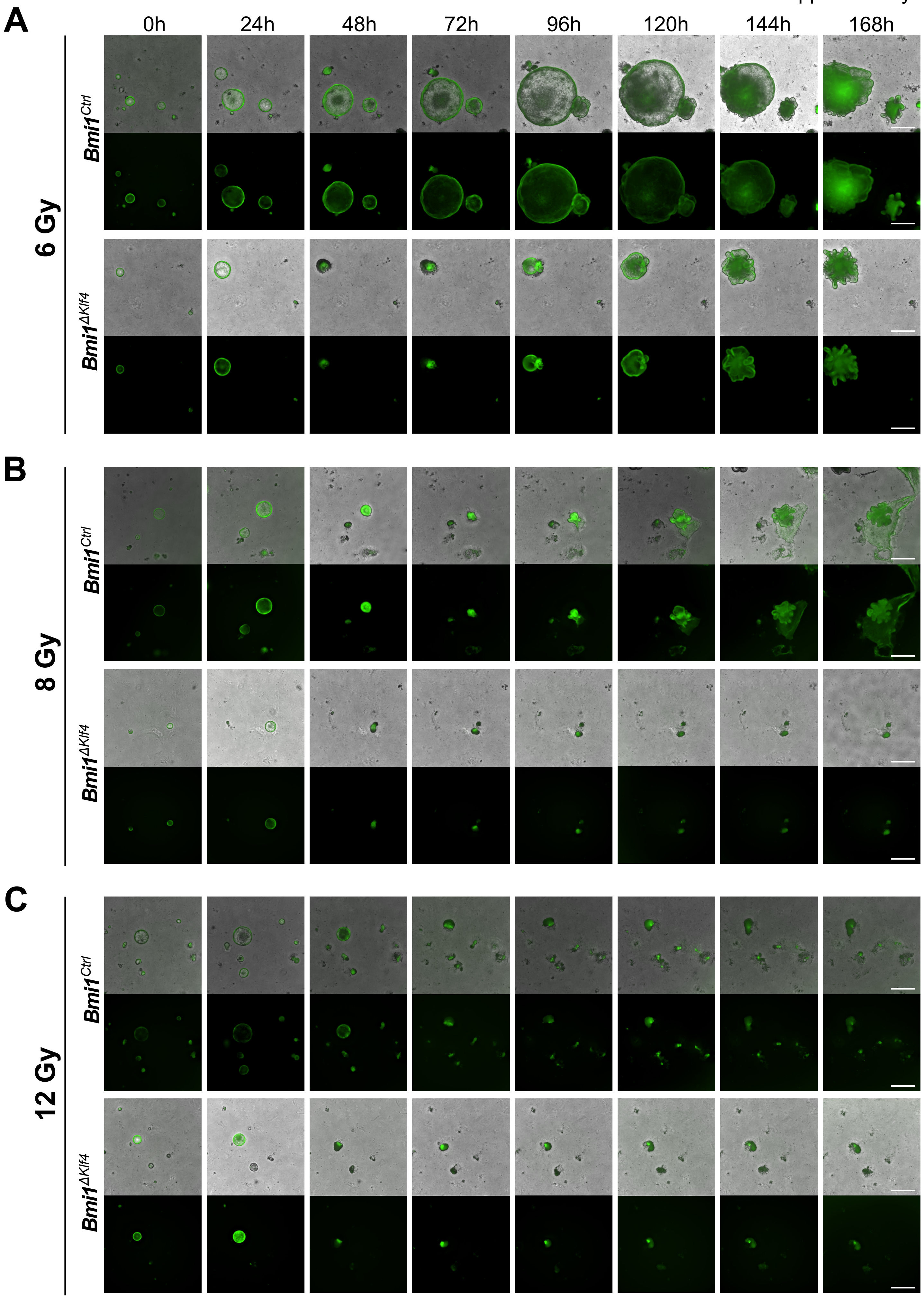

Supplementary Figure 9. KLF4 influences the YFP$^+$-derived organoid formation and regenerative capability in response to γ radiation-induced injury. (A-C) Representative images of organoids derived from the FACS-sorted YFP$^+$ cells isolated from the *Bmi1*^{Ctrl}and *Bmi1*$^{\Delta Klf4}$ mice at 0, 6, 24, 48, 72, 96, 120, 144 and 168 h after irradiation exposed to 6 Gy (A), 8 Gy (B) or 12 Gy (C) source of γ irradiation obtained under a fluorescence microscope. The lower panel represents fluorescent images, the upper panel represents merged images of bright-field and fluorescent images. The scale bar represents 500 µm.

A

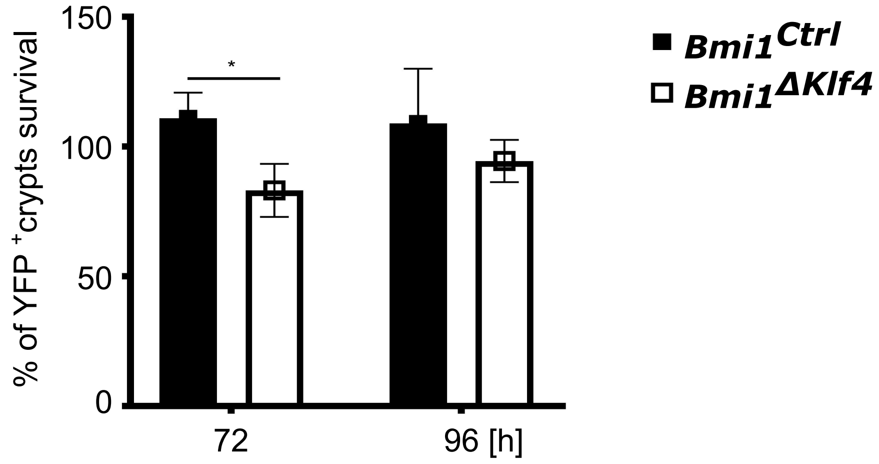

Supplementary Figure 10. Quantification of the YFP^{+} crypts survival 72 h and 96 h post-irradiation of the *Bmi1*^{Ctrl} and *Bmi1*$^{\Delta Klf4}$mice treated according to protocol 1 (Supplementary Fig. 1A) presented as the percentage of the YFP^{+} proliferating crypts after irradiation *vs.* sham. Data are represented as the mean ± SD, 200 YFP^{+} crypts were quantified per mouse, and n = 3 mice per group. * $p < 0.05$ by Student's t-test.

Uncropped blots in Fig. 3

Fig. 3C

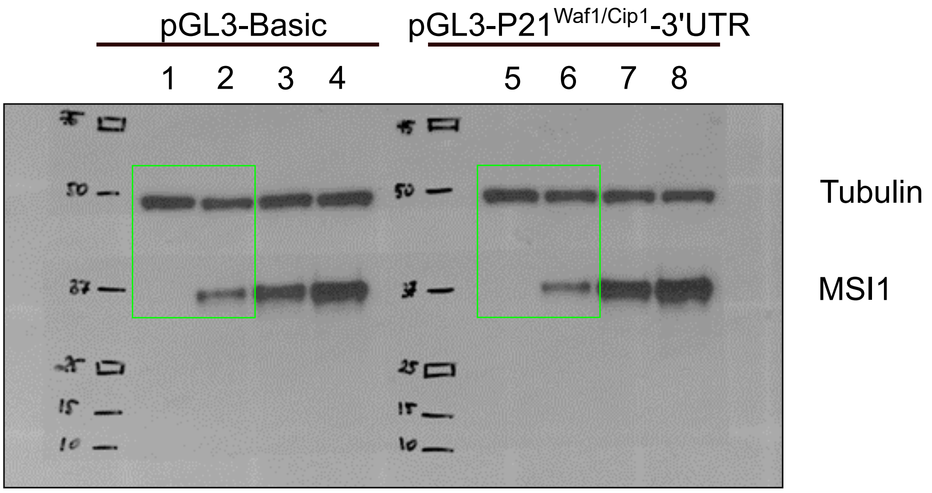

HEK293T cells were transfected with 100 ng of pGL3-Basic (lanes 1-4) or pGL3-P21$^{Waf1/Cip1}$-3'UTR (lanes 5-8). Additionally cells were transfected with 100 ng pReceiver-Lv216 (EV; lanes 1 and 5) or pCDH-CMV-Msi1 100 ng (lanes 2 and 6), 150 ng (lanes 3 and 7), and 200 ng (lanes 4 and 8). Protein ladder was marked.

Fig. 3H

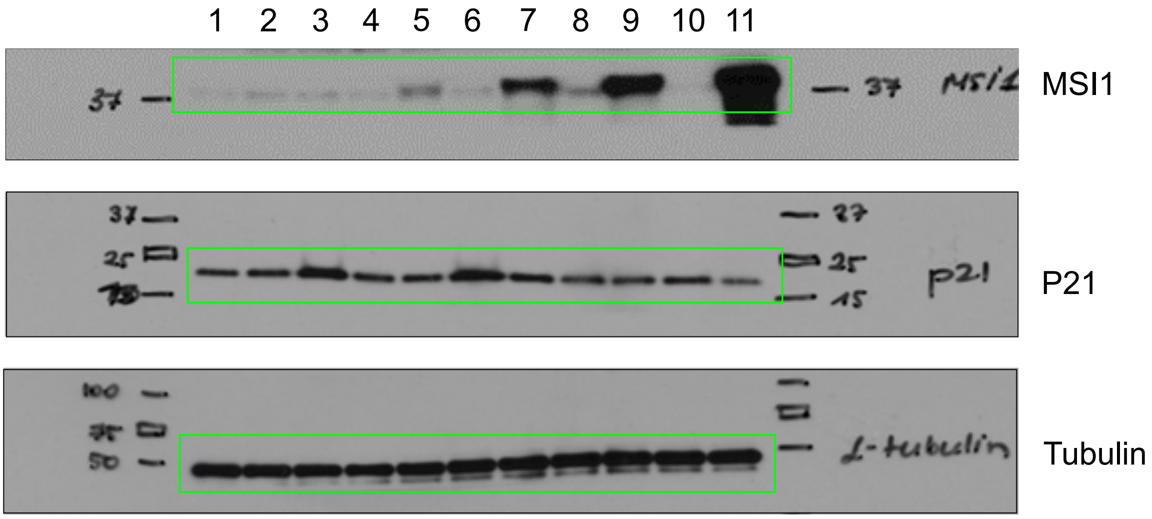

Lane	1	2	3	4	5	6	7	8	9	10	11
12 Gy	-	-	+	-	-	+	+	-	-	+	+
MSI1 O/E	-	-	-	-	+	-	+	-	+	-	+

HEK293T cells were irradiated with dose of 12 Gy or remained non-treated and 24 h later MSI1 was expressed using 100 ng of pCMV6-AC-GFP-MSI1 encoding human MSI1. Samples without MSI1 overexpression were transfected with 100 ng of pcDNA3.1 used as an empty vector (EV) control.

Uncropped blots in Fig. 5

Fig. 5B

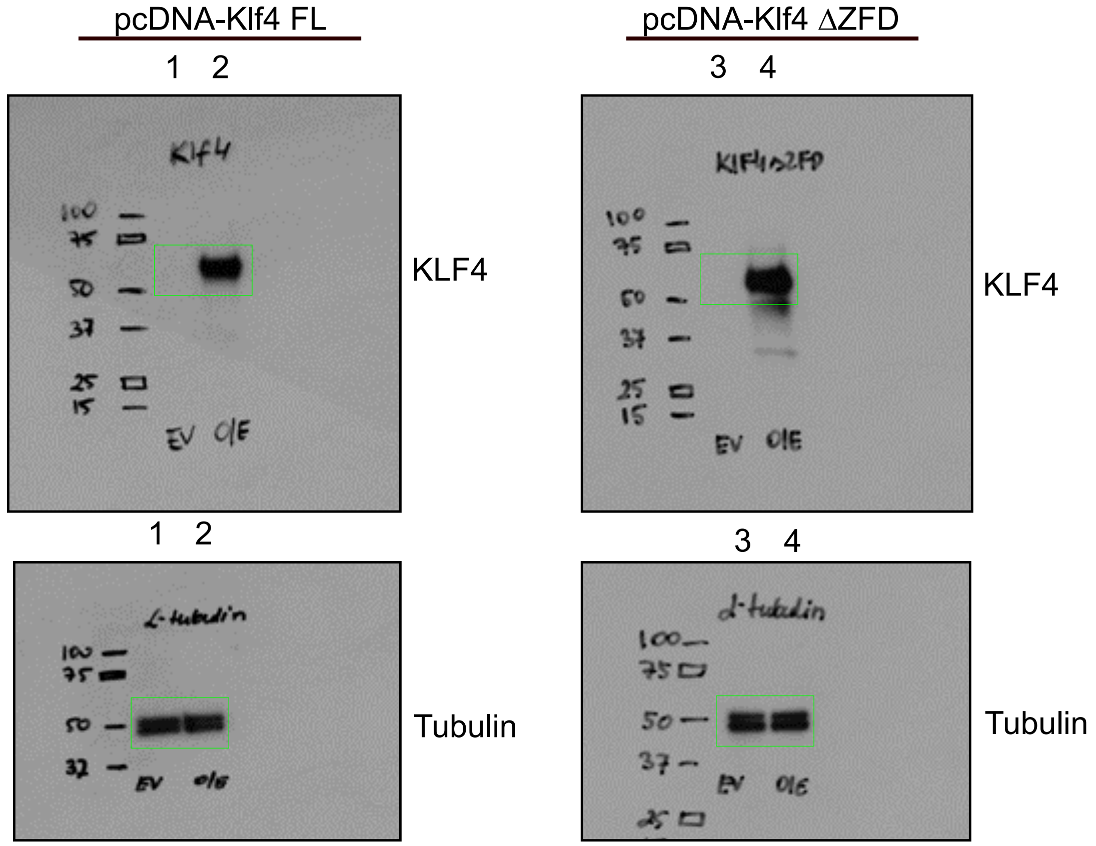

HEK293T cells were transfected with 100 ng of pcDNA3.1 (EV, empty vector control, lanes 1 and 3) or 100 ng of pcDNA3.1-Klf4 FL coding mouse full length *Klf4* (lane 2) or pcDNA3.1-Klf4 DZFD coding mouse *Klf4* mutant with deletion of C-terminal DNA-binding domains and encoding aminoacids from 1 to 349 of the full length protein (lane 4).

Uncropped blots in Fig. 5

Fig. 5F

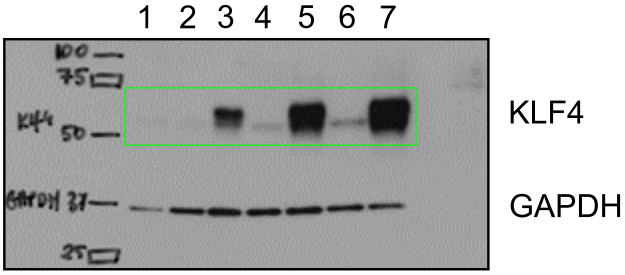

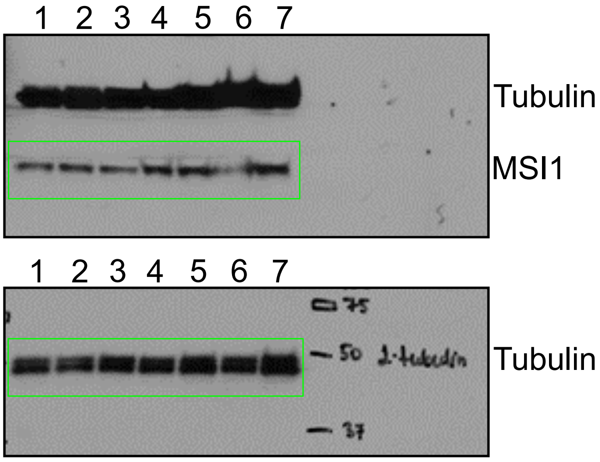

Lane	1	2	3	4	5	6	7
Time [h]	0	24	24	48	48	72	72
KLF4 O/E	-	-	+	-	+	-	+

HEK293T cells were transfected with 100 ng of pcDNA3.1 (EV, empty vector control, lanes 1, 2, 4 and 6) or 100 ng of pcDNA3.1-KLF4 FL coding human full length *KLF4* (lanes 3, 5 and 7). For tubulin both shorter and longer expositions are shown.

Uncropped gels in Fig. 5

Fig. 5I

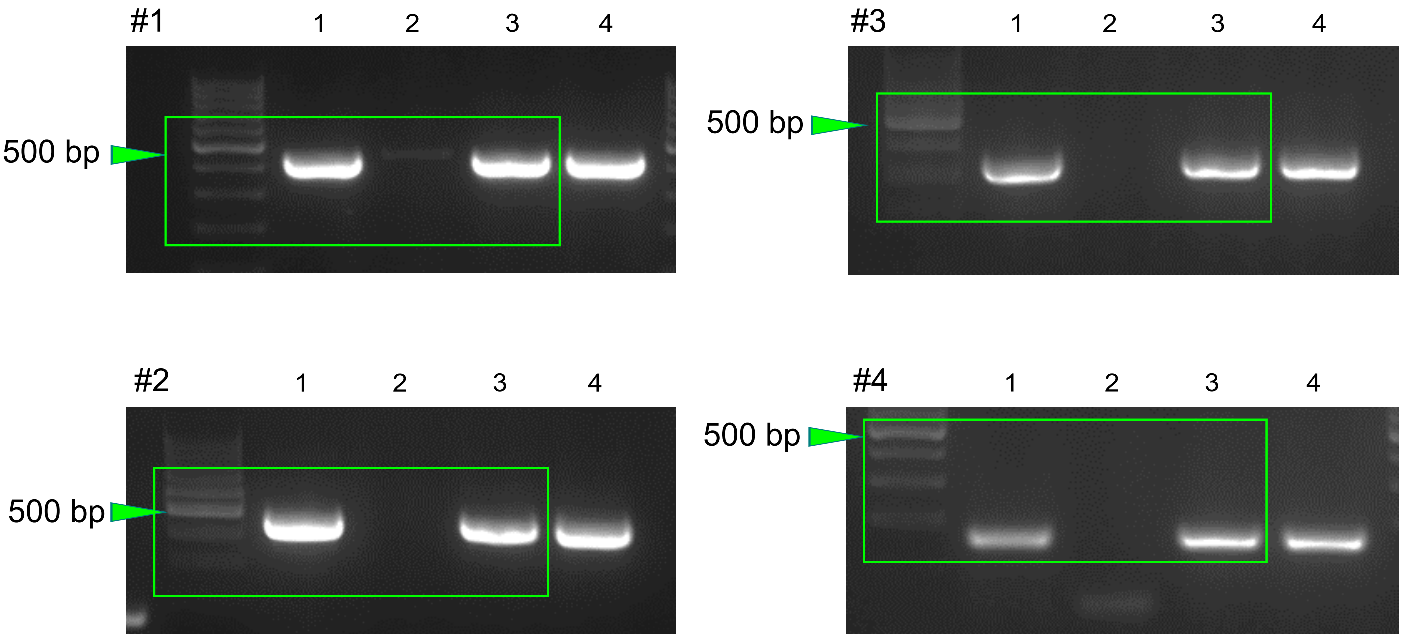

Lane:
1. input
2. IgG
3. KLF4 Ab 10 μL
4. KLF4 Ab 15 μL
